# Nanoparticle Analysis for Reference Materials: The Small-Angle X‑ray Scattering Method

**DOI:** 10.1021/acs.langmuir.6c03792

**Published:** 2026-09-23

**Authors:** Andreas F. Thünemann

**Affiliations:** 42220 Bundesanstalt für Materialforschung und -prüfung (BAM), Unter den Eichen 87, 12205 Berlin, Germany

## Abstract

Reliable certification of nanoparticle reference materials is crucial for advancing nanoparticle analysis. Here, we demonstrate the use of small-angle X-ray scattering (SAXS) as a robust method for certifying a silver nanoparticle reference material (BAM-N008) exhibiting exceptional long-term stability—maintaining its properties for at least 10 years without special storage precautions. Comprehensive characterization in accordance with ISO 17867 and ISO 23484 supports the suitability of SAXS for nanoparticle metrology. Homogeneity and stability studies underpin value assignment and uncertainty evaluation, with metrologically traceable values established for particle diameter and number density, and reference values assigned for the width of the size distribution and particle mass concentration. The assigned values are *D* = 5.8 nm, σ = 1.33 nm, *N* = 3.7 × 10^–6^ mol L^–1^, and *c* = 3.0 g L^–1^, with expanded uncertainties (*k* = 2) of 9%, 14%, 19%, and 17%, respectively. These results provide a reliable basis for validating and verifying analytical methods for nanoparticle dispersion analysis.

## Graphical abstract

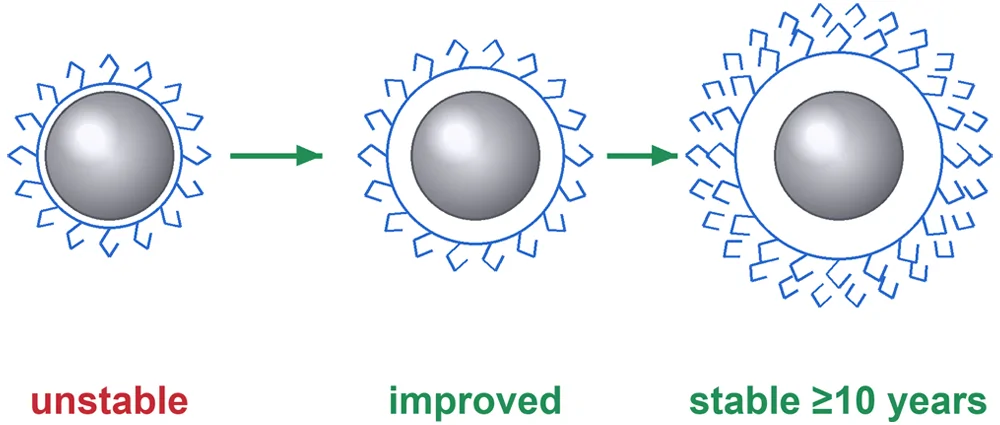

## Introduction

The applications of silver nanoparticles are remarkably diverse, ranging from antimicrobial agents to performance enhancers in photovoltaic systems.^1^ Consequently, substantial research efforts have been devoted to both the development of novel applications and the assessment of their long-term environmental, health, and safety implications.^2^ From the outset, concerns have persisted regarding their safety and environmental impact.^3^ The distinct behavior of nanoparticles relative to bulk materials arises from their reduced dimensions and high surface-to-volume ratio, which result in a large fraction of atoms residing at the particle–environment interface. These characteristics, however, also confer limited thermodynamic stability. In particular, silver nanoparticle suspensions are susceptible to aging during storage. For example, Gorham et al. reported that citrate-capped silver nanoparticles progressively lose their physical and chemical integrity through aggregation, agglomeration, and oxidation.^4^ To mitigate these effects, storage under nitrogen and in the absence of light has been recommended. Aging can significantly alter the toxicity of coated silver nanoparticles via dissolution, agglomeration, and degradation of the capping agent.^5^ Consistent with this observation, the reduced thermodynamic stability and consequent agglomeration of nanoparticles over time can, as a simplified explanation, be attributed to their high specific surface area.^6^ Accordingly, continued investigation of silver nanoparticle-containing materials, including their production, application, environmental distribution, and effects on human health and ecosystems, remains essential.^7^ Reliable characterization methods are critical in this context. A state-of-the-art work on nanoparticle size and concentration analysis has been highlighted in recent interlaboratory comparisons.^8^ Here, comparability is understood with respect to a clearly defined measurand, namely the size and size distribution of the metallic core; for core–shell particles such as the poly­(acrylic acid)-stabilized silver nanoparticles studied here, SAXS and dynamic light scattering (DLS) probe different measurands (core diameter versus hydrodynamic diameter, respectively) and should therefore be regarded as complementary rather than directly comparable techniques. To ensure traceability of measurements and comparability across analytical techniques, the use of reference materials (RMs) and certified reference materials (CRMs) is indispensable.^9^ The objective of this study is twofold: to provide a silver nanoparticle reference material that is straightforward to prepare and exhibits exceptional long-term colloidal stability, and to demonstrate, through a complete ISO 17867/23484-conformant homogeneity and stability study, that SAXS provides a robust, metrologically traceable basis for certifying such a material. The certification of the silver nanomaterial was carried out using small-angle X-ray scattering (SAXS) measurements, employing a validated method that provides fully quantified and traceable total measurement uncertainties.^10–12^ SAXS is a well-established technique for the metrologically traceable determination of both the diameter and the number concentration of spherical nanoparticles.^12–14^ As part of the development of this reference material, we conducted an international interlaboratory comparison of SAXS measurements as a feasibility study.^15^ The material examined in that study was a suspension of silver nanoparticles prepared using the same protocol as that adopted here for BAM-N008. The interlaboratory comparison demonstrated reliable determination of the measurands: mean particle diameter (*D*), width of the size distribution (σ), particle number concentration (*N*), and mass concentration (*c*). Therefore, the selection of measurands for the present study was guided by the outcomes of that interlaboratory exercise. Throughout this work, small-angle X-ray scattering was consistently used to assess the material’s homogeneity and stability and to provide comprehensive characterization. Achieving target uncertainties of 5% for *D*, 10% for σ, and 20% for both *N* and *c* is regarded as a desirable objective.

## Experimental Section

### Materials

Water (Milli-Q, 18.2 MΩ cm at 25 °C) was used for all preparations. All chemicals were used as received without further purification. Silver nitrate was obtained from AppliChem, ethylene glycol from Acros, and poly­(acrylic acid) (PAA) from Fluka (catalog no. 323667-250 g).

### Synthesis of Silver Nanoparticles

Silver nanoparticles were synthesized via a modified polyol process based on literature procedures.^16^ We reported the details earlier.^17^ We describe the synthesis here to facilitate reproducibility. Solution 1 was prepared by dissolving 1.944 g (11.444 mmol) of silver nitrate in 58.5 mL of ethylene glycol under stirring at room temperature. Solution 2 was prepared by dissolving 15.7 g (217.9 mmol, with respect to monomer units) of PAA in 291.5 mL of ethylene glycol, followed by heating to 200 °C under stirring. A short-chain PAA (*M*_w_ = 1800 g mol^–1^, corresponding to approximately 25 monomer units per chain) was used. The molar ratio of Ag^+^ to acrylic acid units was 1:19. Solution 1 was injected rapidly into the boiling Solution 2 within 3 s under vigorous stirring. The mixture was refluxed for 15 min and then allowed to cool to room temperature. For workup, 800 mL of water (pH 5) was added, and the dispersion was left to stand for 24 h. The reddish-brown supernatant was then decanted. This washing step was repeated three times. The final residue, from which ethylene glycol had been removed by the repeated water-washing steps described above, was redispersed in 150 mL of water to obtain the aqueous dispersion used in all subsequent processing and SAXS measurements. A 1% (w/v) NaOH solution was added dropwise until the pH reached 10, resulting in a color change from reddish-brown to olive green, then to brownish-black. Thermogravimetric analysis (TGA) showed a PAA content of 13 wt % relative to the dry mass after drying the samples at 80 °C. Additional PAA (0.35 g L^–1^) was then added, yielding a final PAA content of 30 wt %, as confirmed by TGA. TGA curves of similar samples have been reported previously (see Figure 1 in Kästner and Thünemann^17^). A total of 19 batches were prepared at BAM between May and August 2016, following the procedure described above. The batches were combined in a 10 L amber glass vessel to obtain 7 L of dispersion. The resulting stock dispersion was stored at 20 ± 3 °C.

**Figure 1:**
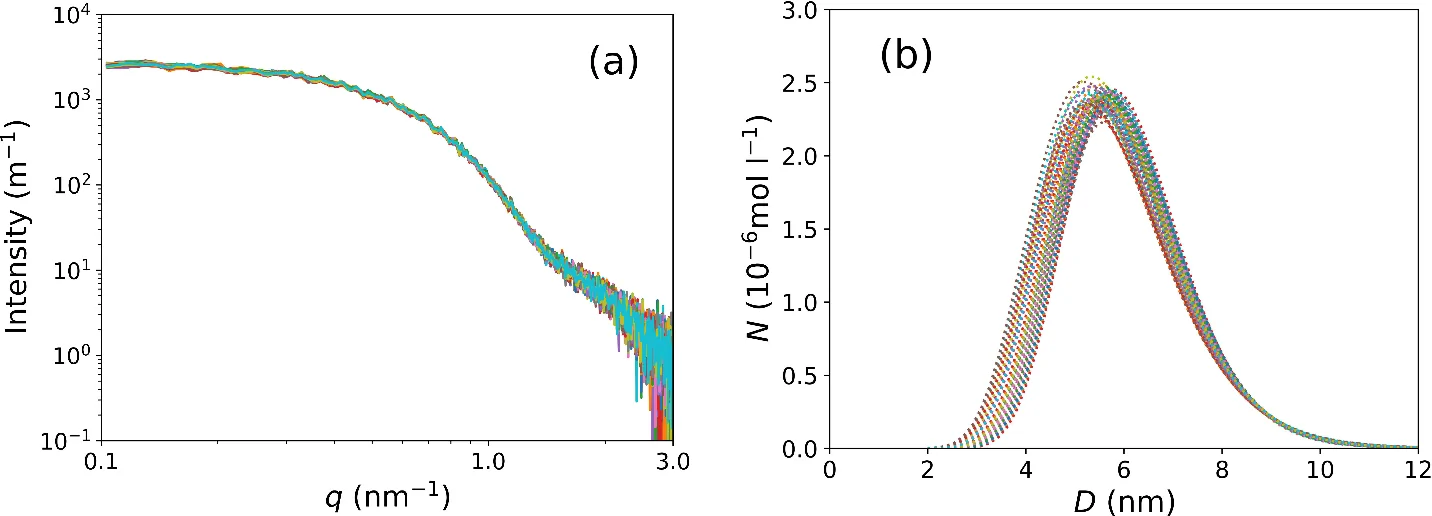
(a) Overlay of the 60 SAXS data sets obtained in the homogeneity and stability study. The scattering intensity is reported on an absolute scale, [*I*] = m^–1^, using water as the calibration standard according to Orthaber et al.^19^ (b) Overlay of 60 number-weighted probability density functions (PDFs) of the particle diameters obtained from the SAXS data in panel (a).

### Preparation of the Reference Material

The stock dispersion contained approximately 3 g L^–1^ of silver. The suspension was aliquoted into 5 mL portions using a bottle-top dispenser (DispensetteIII, digital, 1–10 mL) and filled into amber high-density polyethylene (HDPE) bottles (Thermo Scientific Nalgene, nominal volume 8 mL). The lot comprised 1000 bottles, with an example shown in Figure S1. The amber HDPE material reduces UV light transmission and thus protects light-sensitive samples. It also provides excellent chemical resistance toward acids, bases, and alcohols, ensuring long-term stability during storage and transport. The bottles were sealed with leak-tight screw caps. The pH of the dispersion was routinely monitored. At the time of delivery, the pH was approximately 10, although slight variations are not critical for the material properties. The sealed, air-tight bottles preclude exposure to ambient oxygen, so that oxidative or hydroxide formation at the particle surface is expected to be suppressed, consistent with the assumption of a metallic silver core in the SAXS model.

### Small-Angle X-ray Scattering

For material characterization, three independent SAXS reference instruments were utilized. The first instrument, located at BAM, was a SAXSess system (Anton Paar AG, Austria). This Kratky-type SAXS instrument was coupled with a laboratory X-ray generator (PW3830, PANalytical), operating with a fine-focus glass X-ray tube at 40 kV and 40 mA. The measurements were performed using Cu K_α_ radiation (λ = 0.15418 nm). A focusing multilayer optic, combined with a block collimator, delivers a monochromatic primary beam with minimal background noise, as detailed by Bergmann et al.^18^

Samples with a volume of 50 μL were introduced into a flow-through plastic capillary with an inner diameter of 1 mm. The flow-through capillary ensured that identical scattering volumes were measured for the sample, the solvent, and the empty capillary. The scattered intensity as a function of the scattering vector *q* was recorded using a Mythen detection system (Dectris AG, Switzerland). The measured intensity data were subsequently converted to absolute values using SAXSquant software (Anton Paar AG), following the method described by Orthaber et al.^19^ Data were collected over a *q*-range from 0.1 to 3 nm^–1^, consistent with the interlaboratory comparison.^15^ Data outside this range were excluded from analysis. The temperature was controlled using a TCS 120 sample holder (Anton Paar AG) with an accuracy of ±1 °C. The scattering curves obtained for the homogeneity study are presented in Figure 1a. The second instrument operated at BAM was a Nano-inXider (Xenocs, Grenoble, France). The third instrument was the SAXS setup of the Physikalisch-Technische Bundesanstalt (PTB, Berlin, Germany), located at the Helmholtz-Zentrum Berlin für Materialien und Energie (HZB) and installed at the PTB four-crystal monochromator beamline.^12^ The sample was injected into a glass capillary with an inner diameter of 1 mm and a wall thickness of 10 μm. Scattering patterns were recorded over 3600 s using a charge-coupled device (CCD)-based detector at a photon energy of 10 keV. The sample-to-detector distance was 2.561 m. The transmitted beam was blocked by a rectangular beam stop positioned in front of the detector. A scattering pattern obtained from a water-filled capillary was used for background correction. The intensity values measured with instrument three were not scaled to absolute intensity. The SAXS data were evaluated in accordance with the international standards ISO 17867 and ISO 23484,^13,14^ utilizing a model based on a lognormal size distribution of spherical particles. An example of this model’s application is shown in Figure 2, which displays a representative data set from the homogeneity study (measurement identifier S9602, sample ID0970). The resulting fits provide estimates of the silver nanoparticle diameter distributions, presented in Figure 1b. A detailed description of the data evaluation procedure is given in the Theory section. The minimum sample volume for reliable determination of particle properties should be chosen to avoid significant sample heterogeneity. For SAXS measurements, however, the sample volume is primarily determined by the capillary’s dimensions and capacity, and no strict requirement therefore applies. As a general guideline, at least 50 μL of the suspension should be used for a single analysis. Although this aspect was not explicitly investigated in the present work, it is reasonable to assume that smaller sample volumes would yield comparable results. This assumption is supported by previous studies on certified reference materials of gold nanoparticles, where sample volumes as small as 20 μL and 3 μL were successfully employed.^20^

**Figure 2:**
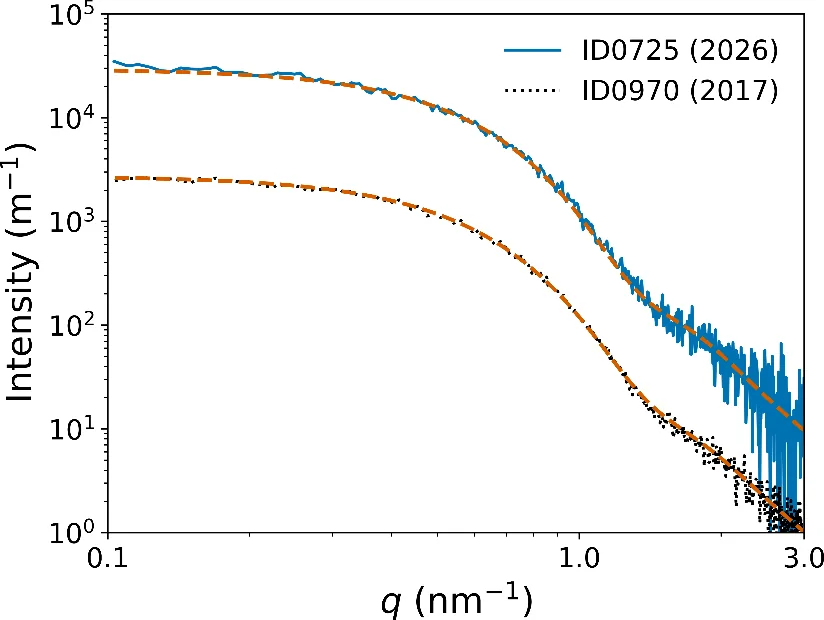
Representative SAXS data analyzed using the model in eq 1 for a sample from the 2017 homogeneity study (ID0970) and a second sample measured in 2026 (ID0725), shown as black and blue curves, respectively. The scattering intensity is plotted as a function of the scattering vector *q*, together with the corresponding best fits (red dashed lines). For clarity, the data for sample ID0725 are scaled by a factor of 10.

## Theory

### Model for Interpretation of the SAXS Data

A model-based evaluation of small-angle X-ray scattering data is performed to characterize the size distribution and concentration of the silver nanoparticles. The SAXS model for non-interacting spherical particles is applied in accordance with ISO 17867,^13^ assuming a constant internal particle density and a homogeneous continuous dispersing phase. The model is expressed as

1 $$I\left(q\right)=N_{n}{\left(\rho_{2}-\rho_{1}\right)}^{2}\int_{0}^{\infty }f\left(r\right){\left[\frac{4\pi }{3}r^{3}\right]}^{2}P\left(q,r\right)dr$$

where *I*(*q*) is the scattering intensity, *q* is the scattering vector, *N*_*n*_ is the particle number density, ρ_2_ is the scattering length density of the particles (silver), and ρ_1_ is the scattering length density of the continuous phase (water).

The ρ values are calculated using the scattering length density utility of the SASfit software.^21^ For silver, a value of ρ_2_ = 7.738 × 10^11^ cm^–2^ is obtained by assuming a density of 10.49 g cm^–3^. The bulk density of metallic silver is used as an approximation for the SAXS contrast calculation, because Bragg reflections of particles in this size range are substantially broadened and a large fraction of atoms reside in a disordered near-surface region, such that a diffraction-based crystallinity assessment would itself only provide a rough estimate. The Cu K_α_ radiation energy is 8047.60 eV. For water, a value of ρ_1_ = 9.446 × 10^10^ cm^–2^ is calculated using a density of 0.998 g cm^–3^. Accordingly, a scattering length density contrast of ρ_2_ – ρ_1_ = 6.793 × 10^11^ cm^–2^ is employed. The scattering vector is defined as *q* = 4πλ^–1^ sin θ, where λ = 0.15418 nm is the X-ray wavelength and θ is the scattering angle.

The function *f*(*r*) denotes the number-weighted particle radius distribution, and *P*(*q*,*r*) is the corresponding form factor, given by

2 $$P\left(q,r\right)={\left[3\frac{\sin\left(qr\right)-qr\cos\left(qr\right)}{{\left(qr\right)}^{3}}\right]}^{2}$$

In this study, *f*(*r*) is described by a lognormal distribution, as recommended in ISO 17867 ^13^ and previously applied successfully in an interlaboratory comparison for the determination of particle size distributions by SAXS.^15^ We often encounter the argument that assuming a Gaussian rather than a lognormal distribution can lead to significantly different results. In this context, it should be noted that this is not the case for sufficiently narrow distributions, as demonstrated by the consistency of results obtained using different evaluation approaches in SAXS interlaboratory comparisons.^15^ In particular, deviations remain small as long as the width of the distribution does not exceed approximately 20% of the mean value. The lognormal size distribution is defined as

3 $$f\left(R\right)=\frac{1}{\sqrt{2\pi }wR}\exp\left[-\frac{\ln^{2}\left(R/R_{\mathrm{median}}\right)}{2w^{2}}\right]$$

where *R*_median_ denotes the median particle radius and *w* characterizes the distribution width. It should be noted that *w* represents the standard deviation of the natural logarithm of the radius distribution (dimensionless), and should not be confused with the standard deviation of the radius distribution itself, which has units of nanometers (nm). Because the lognormal distribution is fully defined by *R*_median_ and *w*, additional parameters can be derived from these quantities. Common derived parameters include the mean, mode, and standard deviation. The mean radius is given by

4 $$R_{\mathrm{mean}}=R_{\mathrm{median}}\exp\left(\frac{w^{2}}{2}\right)$$

with an associated uncertainty of

5 $$u_{R_{\mathrm{mean}}}={\left[e^{w^{2}}\left(u_{R_{\mathrm{median}}}^{2}+{\left(R_{\mathrm{median}}wu_{w}\right)}^{2}\right)\right]}^{1/2}$$

where the uncertainties *u*_*R*_median__ and *u*_*w*_ are obtained from the fitting procedure (see^21^ for details). The width of the radius distribution is given by

6 $$\sigma_{\mathrm{lognormal}}={\left[e^{2w^{2}}-e^{w^{2}}\right]}^{1/2}R_{\mathrm{median}}$$

with an associated uncertainty of

7 $$u_{\sigma_{\mathrm{lognormal}}}={\left(\left[e^{2w^{2}}-e^{w^{2}}\right]u_{R_{\mathrm{median}}}^{2}+\frac{e^{w^{2}}{\left(1-2e^{w^{2}}\right)}^{2}R_{\mathrm{median}}^{2}w^{2}}{e^{w^{2}}-1}u_{w}^{2}\right)}^{1/2}$$

We define *N*_fit_ = *N*_*n*_(ρ_2_ – ρ_1_)^2^ as an effective scaling factor to avoid numerically unfavorable handling of very large values of *N*_*n*_ and very small values of (ρ_2_ – ρ_1_)^2^ during the curve fitting procedure. Together with the abbreviation

8 $$I_{\mathrm{fit}}\left(q\right)=\int_{0}^{\infty }f\left(r\right){\left[\frac{4\pi }{3}r^{3}\right]}^{2}P\left(q,r\right)dr$$

the scattering intensity can be written as

9 $$I\left(q\right)=N_{\mathrm{fit}}I_{\mathrm{fit}}\left(q\right)$$

Here, *I*_fit_(*q*) represents the *q*-dependent contribution in the fitting procedure and yields the parameters *R*_median_ and *w*. The particle diameter is calculated from eq 4 as

10 $$D=2R_{\mathrm{mean}}$$

with an associated uncertainty of

11 $$u_{D}=2u_{R_{\mathrm{mean}}}$$

The width of the diameter distribution is derived from eq 6 as

12 $$\sigma =2\sigma_{\mathrm{lognormal}}$$

with an associated uncertainty of

13 $$u_{\sigma }=2u_{\sigma_{\mathrm{lognormal}}}$$

### Number Density of Particles

To convert the scaling factor *N*_fit_ into the particle number density *N*_*n*_ (particles per cm^3^), it is essential to account for the units of the absolute scattering intensity *I*(*q*), the scattering vector *q*, and the scattering length densities ρ_2_ and ρ_1_. From the model employed, it follows that

14 $$N_{\mathrm{fit}}=\frac{I\left(q\right)}{I_{\mathrm{fit}}\left(q\right)}$$

The measured scattering intensity has the unit [*I*(*q*)] = m^–1^, whereas the fitted intensity term has the unit [*I*_fit_(*q*)] = nm^6^. Accordingly, the unit of *N*_fit_ is

15 $$\left[N_{\mathrm{fit}}\right]=\left[I\left(q\right)\right]{\left[I_{\mathrm{fit}}\left(q\right)\right]}^{-1}$$

16 $$=m^{-1}{\mathrm{nm}}^{-6}$$

The particle number density is given by

17 $$N_{n}=\frac{N_{\mathrm{fit}}}{{\left(\rho_{2}-\rho_{1}\right)}^{2}}$$

where the squared scattering length density contrast has the unit [(ρ_2_ – ρ_1_)^2^] = cm^–4^. Thus, the unit of *N*_*n*_ becomes

18 $$\left[N_{n}\right]=\frac{\left[N_{\mathrm{fit}}\right]}{\left[{\left(\rho_{2}-\rho_{1}\right)}^{2}\right]}$$

19 $$=\frac{m^{-1}{\mathrm{nm}}^{-6}}{{\mathrm{cm}}^{-4}}$$

20 $$=10^{40}{\mathrm{cm}}^{-3}$$

This implies that the calculated values of *N*_fit_/(ρ_2_ – ρ_1_)^2^ must be multiplied by a factor of 10^40^ to obtain particle number densities *N*_*n*_ in units of cm^–3^. The uncertainty of *N*_*n*_ is determined by the uncertainties associated with *N*_fit_, ρ_2_, and ρ_1_. The uncertainty in *N*_fit_ can be estimated from the curve-fitting procedure as *u*_*N*_fit__. In contrast, reliable uncertainty estimates for ρ_2_ and ρ_1_ are not available. Therefore, the standard uncertainty, evaluated according to the GUM,^22^ is approximated as

21 $$u_{N_{n}}=\frac{u_{N_{\mathrm{fit}}}}{{\left(\rho_{2}-\rho_{1}\right)}^{2}}$$

In chemistry, concentrations are typically expressed as molar concentrations. The molar concentration is calculated from the particle number density according to

22 $$N=\frac{N_{n}}{N_{A}}$$

with the associated uncertainty given by

23 $$u_{N}=\frac{u_{N_{n}}}{N_{A}}$$

where *N*_A_ denotes the Avogadro constant, *N*_A_ = 6.02214076 × 10^23^ mol^–1^. The corresponding units are

24 $$\left[N\right]=\frac{\left[N_{n}\right]}{\left[N_{A}\right]}$$

25 $$={\mathrm{mol cm}}^{-3}$$

26 $$=10^{3}{\mathrm{mol L}}^{-1}$$

Accordingly, the values of *N*_*n*_ and *u*_*N*_*n*__ must be multiplied by 10^3^/*N*_A_ to convert particle number densities in units of cm^–3^ into molar concentrations in units of mol L^–1^.

### Mass Concentration

The particle mass concentration *c*, expressed in units of [*c*] = g cm^–3^, is calculated as

27 $$c=N_{n}\rho \left\langle V\right\rangle$$

where *N*_*n*_ is the particle number density, ρ is the particle density, and ⟨*V*⟩ is the mean particle volume. Uncertainties can be estimated for *N*_*n*_ and ⟨*V*⟩ from the curve-fitting parameters, whereas no reliable uncertainty estimate is available for ρ, the density of the silver nanoparticles. In this work, the bulk density of silver, 10.49 g cm^–3^, is used for ρ. The combined standard uncertainty, calculated according to the GUM,^22^ is given by

28 $$u_{\mathrm{con}}=\sqrt{u_{1}^{2}+u_{2}^{2}}$$

with *u*_1_ = *ρ*⟨*V*⟩*u*_*N*_*n*__, representing the contribution from the uncertainty of *N*_*n*_, and *u*_2_ = *N*_*n*_ρ*u*_⟨*V* ⟩_, representing the contribution from the uncertainty of the mean particle volume ⟨*V*⟩, which is determined by the particle size distribution. For spherical particles,

29 $$\left\langle V\right\rangle =\frac{4}{3}\pi \left\langle R^{3}\right\rangle$$

so the distribution of particle radii must be accounted for. For a lognormal radius distribution, the mean particle volume is

30 $$\left\langle V\right\rangle =\frac{4\pi }{3}R_{\mathrm{median}}^{3}\exp\left(\frac{9w^{2}}{2}\right)$$

where *R*_median_ is the median particle radius and *w* is the standard deviation of the logarithm of the radius. The combined standard uncertainty of ⟨*V*⟩ is

31 $$u_{\left\langle V\right\rangle }=\sqrt{u_{21}^{2}+u_{22}^{2}}$$

with the individual contributions given by

32 $$u_{21}^{2}={\left(4\pi R_{\mathrm{median}}^{2}\exp\left(\frac{9w^{2}}{2}\right)\right)}^{2}u_{R_{\mathrm{median}}}^{2}$$

33 $$u_{22}^{2}={\left(12\pi wR_{\mathrm{median}}^{3}\exp\left(\frac{9w^{2}}{2}\right)\right)}^{2}u_{w}^{2}$$

## Results and Discussion

### Assessment of Homogeneity

To assess the uniformity of the silver nanoparticle dispersion, a homogeneity study was conducted. Twenty bottles were randomly selected from the 1000 bottles produced, and one sample from each was analyzed by SAXS on three consecutive days, resulting in a total of 60 measurements. Sample integrity and measurement conditions were assumed constant over the 3 days to ensure comparability. The overlaid 60 SAXS curves allow for direct visual assessment of between-bottle consistency in scattering profiles (Figure 1a). Model fitting of the SAXS data was performed in accordance with ISO 17867,^13^ applying a homogeneous sphere model with a lognormal size distribution (see section Model for Interpretation of the SAXS Data for details). This approach provided key parameters for further statistical evaluation of material homogeneity.

Representative fits are shown in Figure 2, demonstrating good agreement between the model and the experimental data. The corresponding fit parameters were used to derive lognormal size distributions according to eq 3. An overlay of the resulting number-weighted probability density functions (PDFs) of the particle diameters is shown in Figure 1b. The PDFs extend from approximately *D* = 2.5 to 10 nm, with a maximum near 6 nm. While the distributions appear visually consistent, their statistical homogeneity must also be assessed, as discussed in the following section.

Figure 3 summarizes the measurand values for the 20 samples, including the mean and standard deviation of the particle diameter *D*, distribution width σ, particle number density *N*, and concentration *c* (black circles with error bars). The corresponding overall mean values are *D* = (5.83 ± 0.13) nm, σ = (1.33 ± 0.05) nm, *N* = (3.74 ± 0.15) × 10^–6^ mol L^–1^, and *c* = (3.04 ± 0.04) g L^–1^. Red squares indicate the overall mean with one standard deviation, while blue circles represent the overall mean with two standard deviations. An analysis of variance (ANOVA) was performed for all four measurands in accordance with the ISO 33405 ^23^ standard. Data variability was characterized using the between-group mean square, *M*_between_, and the within-group mean square, *M*_within_, which were used to estimate the between-bottle standard deviation,

34 $$s_{bb}=\sqrt{\max\left(\frac{M_{\mathrm{between}}-M_{\mathrm{within}}}{n_{0}},0\right)}$$

where *n*_0_ is the number of observations per group (*n*_0_ = 3 in the present study, corresponding to measurements performed on days 1–3). When *M*_between_ < *M*_within_, *s*_*bb*_ was set to zero. The repeatability standard deviation was calculated as

35 $$s_{r}=\sqrt{M_{\mathrm{within}}}$$

The resulting between-bottle and repeatability standard deviations were *s*_*bb*_ = 0.034 nm and *s*_*r*_ = 0.128 nm for *D*, *s*_*bb*_ = 0 and *s*_*r*_ = 0.054 nm for σ, *s*_*bb*_ = 0.052 × 10^–6^ mol L^–1^ and *s*_*r*_ = 0.146 × 10^–6^ mol L^–1^ for *N*, and *s*_*bb*_ = 0 and *s*_*r*_ = 0.045 g L^–1^ for *c*. The results are summarized in Table 1. The very small, and in some cases zero, values of *s*_*bb*_ indicate that no detectable between-bottle effect was observed for *D*, σ, *N*, or *c*. This finding is consistent with the sample preparation procedure, as all bottles were filled from the same stock suspension, and sedimentation of the nanoparticles is not expected because of their small size.

**Figure 3:**
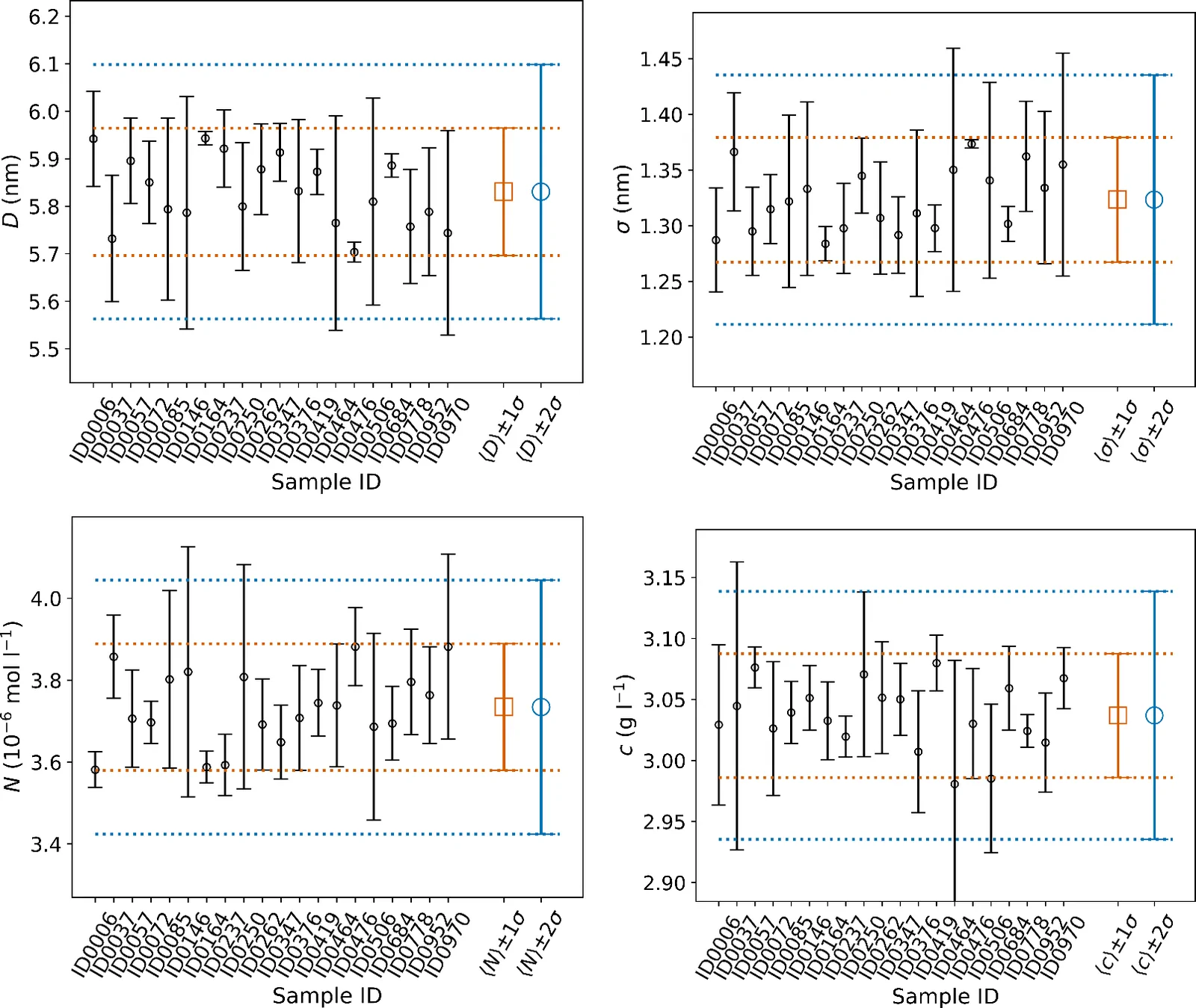
Results of the homogeneity study for the particle diameter *D*, distribution width σ, particle number density *N*, and particle concentration *c*. The 20 samples are identified by their sample IDs. Black circles with error bars represent the mean value and standard deviation for each sample. Red squares with error bars indicate the overall mean ± 1 standard deviation, while blue circles with error bars denote the overall mean ± 2 standard deviations.

**Table 1:** ANOVA Results for the Between-Bottle Homogeneity Study of *D*, σ, *N*, and *c* ^a^

measurand	source	mean	*s*	*SS*	*df*	*MS*	*s*	*F*	*F*_crit_	*p*
*D* (nm)	between	5.827	0.132	0.374	19	0.020	0.034	1.21	1.85	0.298
	within			0.650	40	0.016	0.128			
	total			1.024	59					
σ (nm)	between	1.326	0.054	0.055	19	0.003	0.000	0.98	1.85	0.498
	within			0.117	40	0.003	0.054			
	total			0.171	59					
*N* (10^–6^ mol L^–1^)	between	3.738	0.155	0.560	19	0.029	0.052	1.39	1.85	0.189
	within			0.851	40	0.021	0.146			
	total			1.411	59					
*c* (g L^–1^)	between	3.037	0.044	0.034	19	0.002	0.000	0.89	1.85	0.595
	within			0.080	40	0.002	0.045			
	total			0.113	59					

a *SS*, sum of squares; *df*, degrees of freedom; *MS*, mean square; *s*, estimated standard deviation; *F*, ANOVA test statistic; *F*_crit_, critical *F* value; *p*, significance probability.

ANOVA assesses the equality of group means using a variance-based *F* test. The null hypothesis, that group means are equal, is not rejected when the calculated *F* value is smaller than the critical value, *F*_crit_. As shown in Table 1, *F* < *F*_crit_ for all four measurands, confirming the absence of statistically significant differences between bottles.

Additionally, the *p* values, which decrease as *F* increases, are listed in Table 1 as an alternative measure of significance. The observed *p* values of 0.298 for *D*, 0.498 for σ, 0.189 for *N*, and 0.595 for *c* are all well above the commonly used threshold of 0.05. Accordingly, no statistically significant differences between group means were detected for any of the investigated measurands.

In summary, no evidence was found to reject the hypothesis that the material was sufficiently homogeneous. Under these conditions, the uncertainty contribution associated with between-unit heterogeneity was taken as the between-bottle standard deviation,

36 $$u_{bb}=s_{bb}$$

All data obtained in the homogeneity study were therefore retained. On this basis, overall mean values for the four measurands were determined, together with estimates of the uncertainty contribution *u*_*bb*_ arising from possible undetected inhomogeneity. The repeatability standard deviation was further used as an estimate of the uncertainty associated with the repeatability of a single measurement,

37 $$u_{\mathrm{rep}}=s_{r}$$

### Instrument-Related Bias

Since it is, in principle, possible that Instrument 1 yields systematically biased results, samples of the reference material were also measured using two additional instruments. Details are provided in section 2 of the Supporting Information. The results obtained with Instruments 2 and 3 are consistent with those from the homogeneity study conducted using Instrument 1. These findings indicate that the measurement results are not significantly affected by the specific SAXS instrument used, consistent with the previously reported interlaboratory comparison of silver nanoparticles.^15^

### Short-Term Stability

Short-term stability was evaluated using five samples subjected to temperature extremes (−40, 4, and 70 °C for 7 days) and transportation stress (two intercontinental flights). SAXS analysis after treatment showed that most measurand values remained within the ±2 standard deviation range of the homogeneity study. The results indicate that external influences can induce minor but detectable changes in material properties. Details are provided in section 3.1 of the Supporting Information.

### Long-Term Stability

A long-term stability study was initiated after confirming that changes in the measurands induced by temperature variation and transport were substantially smaller than the target uncertainties. The beginning of the study (*t* = 0) was defined as the first day of the homogeneity study, which was performed on days 354, 355, and 356 after bottling. This comparatively long interval between bottling and the start of the homogeneity study was deliberately chosen to assess whether colloidal stability was sufficient to develop a reference material. The long-term stability study was carried out over 4 years using 30 randomly selected bottles stored under the specified shelf-life conditions, i.e., in the dark at an ambient temperature of 20 ± 3 °C. For each measurement occasion, one bottle was randomly selected from this set. The overall mean values of the measurands were *D* = (5.82 ± 0.14) nm, σ = (1.34 ± 0.05) nm, *N* = (3.77 ± 0.19) × 10^–6^ mol L^–1^, and *c* = (3.07 ± 0.05) g L^–1^. An overview of the measurand values as a function of study time is shown in Figure 4. Measured values are shown as black circles, horizontal red lines indicate the corresponding mean values, and red and blue error bars denote ±1 and ±2 standard deviations, respectively. Visual inspection shows that most values fall within the target uncertainties of 5% for *D*, 10% for σ, and 20% for both *N* and *c*, while all values remain within twice these limits (green dotted and solid lines in Figure 4, respectively).

**Figure 4:**
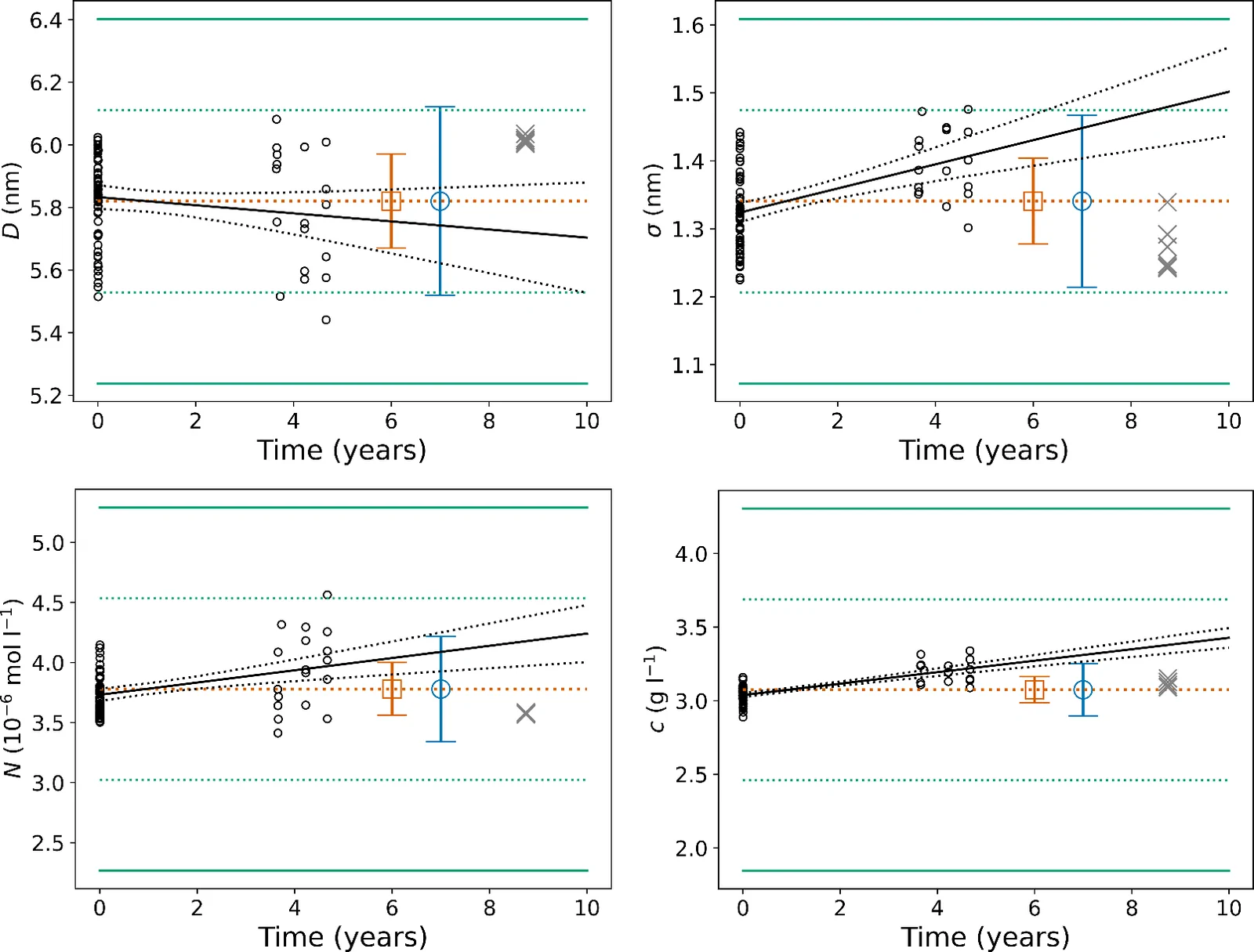
Results of the long-term stability study for particle diameter *D*, size distribution width σ, particle number density *N*, and particle concentration *c*. Measured values are shown as black circles. Dotted red lines indicate the corresponding mean values. Red and blue error bars represent ±1 and ±2 standard deviations of the mean, respectively. Solid black lines show the linear regression fits, with dotted black lines indicating the associated 95% confidence intervals. Horizontal green dotted and solid lines denote one and two times the target uncertainty, respectively. Post-certification measurements are shown as gray crosses.

To evaluate the presence of a time-dependent trend, the rate of change of each measurand was estimated using a univariate linear model in accordance with clause B.3 of the standard ISO 33405:2024.^23^ This approach is described in section 3.3 of the Supporting Information. The resulting regression lines are shown in Figure 4 (black solid lines) over a period of 10 years. The fitted slope is negative for *D* and positive for σ, *N*, and *c*, indicating a decrease in *D* and increases in σ, *N*, and *c* with time. For an estimation of the long-term stability uncertainty contribution, clause 8.7.3 of standard ISO 33405 was applied:^23^

38 $$u_{\mathrm{lts}}=\sqrt{u_{\mathrm{lts},1}^{2}+u_{\mathrm{lts},2}^{2}}$$

with

39 $$u_{\mathrm{lts},1}=s\left(b_{1}\right)\left(t_{m1}+t_{\mathrm{cert}}\right)$$

­(see eq 10 on p 44 of ISO Guide 35) and

40 $$u_{\mathrm{lts},2}=\frac{b_{1}}{2\sqrt{3}}t_{\mathrm{cert}}$$

assuming a rectangular distribution. Here, *t*_m1_ is the time interval between value assignment and the first stability monitoring point (rounded up to 5 years), and *t*_cert_ is the intended shelf life (here 5 years). An overview of the parameters obtained from the long-term stability study is provided in Table 2.

**Table 2:** Parameters Obtained from the Long-Term Stability Study^a^

measurand	*y*_m_	*s*	*u*_t_	*b*_0_	*s*_*b*0_	*b*_1_	*s*_*b*1_	*t*_*b*1_	*t*_95,*n*–2_	*t*_shelf_	*u*_lts_
*D* (nm)	5.82	0.14	0.29 (5%)	5.83	0.02	–0.01	0.01	1.15	1.99	10.85	0.13 (2.26%)
σ (nm)	1.34	0.05	0.13 (10%)	1.33	0.01	0.02	0.00	5.86	1.99	6.07	0.05 (3.47%)
*N* (10^–6^ mol L^–1^)	3.77	0.19	0.76 (20%)	3.73	0.02	0.04	0.01	3.50	1.99	12.00	0.18 (4.65%)
*c* (g L^–1^)	3.07	0.05	0.62 (20%)	3.04	0.01	0.04	0.00	12.16	1.99	14.96	0.04 (1.43%)

a *y*_m_, mean value of the measurand; *s*, standard deviation; *u*_t_, target uncertainty (reported both in the units of the measurand and as a percentage of *y*_m_); *b*_0_, intercept of the linear regression; *s*_*b*0_, standard error of *b*_0_; *b*_1_, slope of the linear regression; *s*_*b*1_, standard error of *b*_1_; *t*_*b*1_, *t* statistic for *b*_1_; *t*_95,*n*–2_, two-tailed critical value of Student’s *t* distribution; *t*_shelf_, predicted shelf life in years; and *u*_lts_, uncertainty contribution due to long-term instability (reported both in the units of the measurand and as a percentage of *y*_m_).

### Measurand Values and Their Uncertainties

The assigned value of each measurand, *y*_char_, was calculated as the arithmetic mean of the 60 data sets, *y*_*i*_, obtained in the homogeneity study:

41 $$y_{\mathrm{char}}=\frac{1}{60}\overset{60}{\underset{i=1}{\sum }}y_{i}$$

The resulting assigned values were *D* = 5.83 nm, σ = 1.33 nm, *N* = 3.74 × 10^–6^ mol L^–1^, and *c* = 3.04 g L^–1^. An overview of the measurand values is presented in Figure 5 as box-and-whisker plots. The red squares denote the mean values, and the red solid lines indicate the assigned values. For all measurands, the whiskers remain within ±2 times the target uncertainty, indicating that the data well support the assigned values.

**Figure 5:**
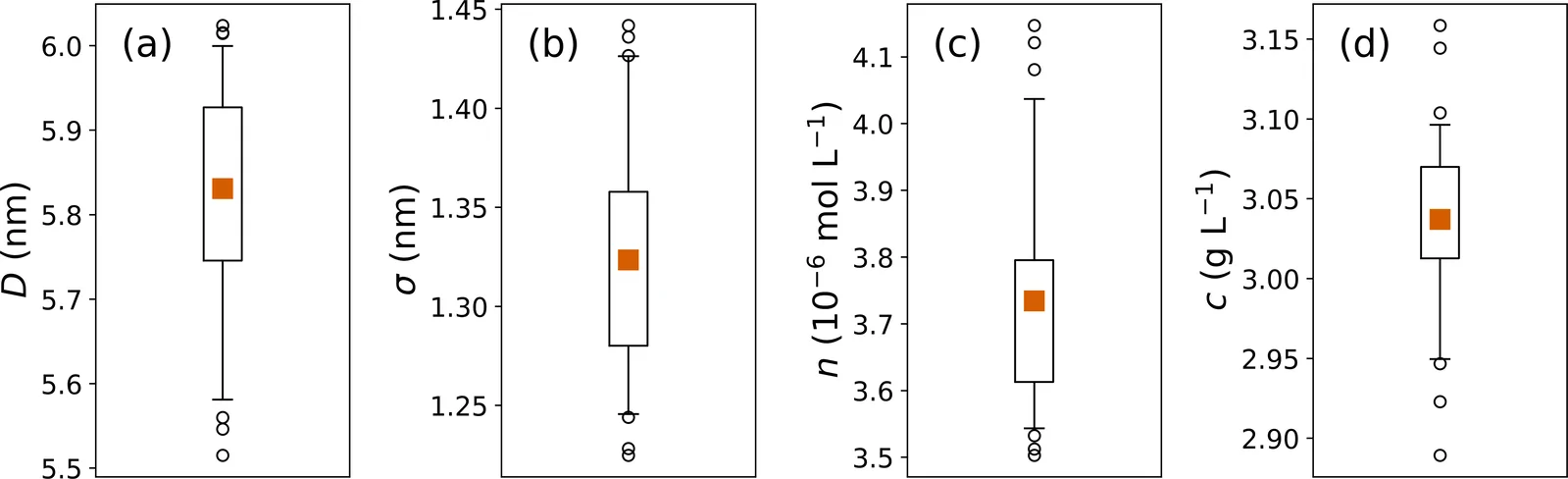
Box-and-whisker plots of the measurands obtained in the homogeneity study. The assigned values are (a) *D* = 5.83 nm, (b) σ = 1.33 nm, (c) *N* = 3.74 × 10^–6^ mol L^–1^, and (d) *c* = 3.04 g L^–1^. Boxes span from the first quartile (*Q*_1_, 25th percentile) to the third quartile (*Q*_3_, 75th percentile), representing the interquartile range, while whiskers extend to the 5th and 95th percentiles. Values outside this range are shown as individual points.

The uncertainty associated with each measurand comprises contributions from several sources. The individual components arising from homogeneity, repeatability, long-term stability, and bias, along with the procedures used to estimate them, are summarized in Table 3. The corresponding uncertainty contributions, *u*_*bb*_, *u*_rep_, *u*_lts_, and *u*_bias_, were derived from the experiments described above and are presented in Figure 6.

**Table 3:** Sources of Uncertainty and Their Estimation

source	symbol	meaning and estimation
homogeneity	*u*_*bb*_	Contribution from between-bottle heterogeneity, estimated from the homogeneity study described in section Assessment of Homogeneity.
repeatability	*u*_rep_	Repeatability contribution for a single measurement, derived from the ANOVA as the average repeatability of the full measurement set (section Assessment of Homogeneity).
stability	*u*_lts_	Contribution associated with possible changes in the measurands over time, estimated from the long-term stability study described in section Long-Term Stability.
bias	*u*_bias_	Contribution associated with possible instrument-related bias, estimated from the comparison with instrument 2 (section Instrument-Related Bias).

**Figure 6:**
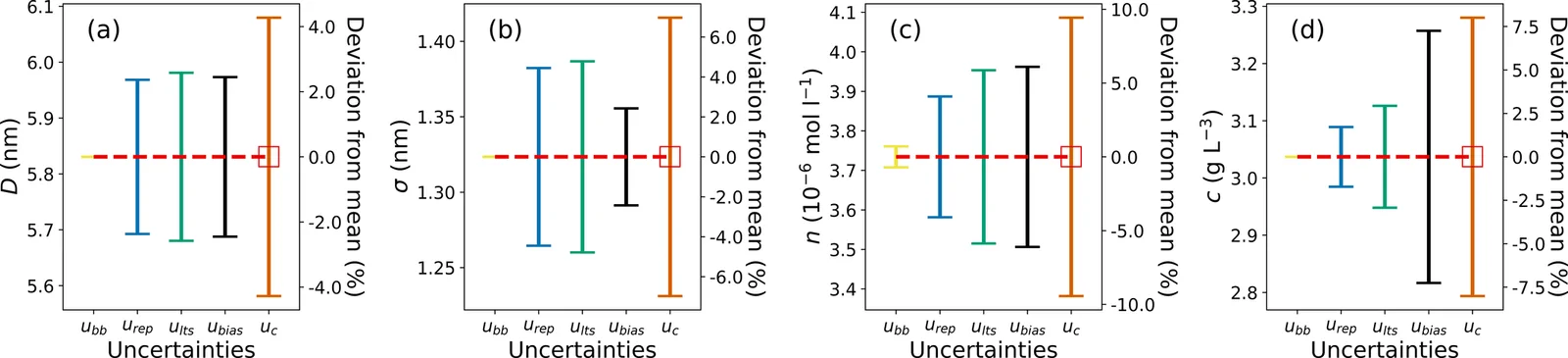
Contributions to the uncertainty of the measurands shown as error bars around the corresponding mean values for (a) particle diameter *D*, (b) distribution width σ, (c) number density *N*, and (d) mass concentration *c*. The individual contributions correspond to *u*_*bb*_, *u*_rep_, *u*_lts_, *u*_bias_, and the combined uncertainty *u*_c_. The red dashed horizontal line indicates the mean value, while the red square marks the corresponding central value for *u*_c_. The secondary right-hand axis shows the deviation from the mean as a percentage.

Among these contributions, *u*_*bb*_ was the smallest, whereas *u*_lts_ was the largest. This behavior is typical of nanoparticles dispersed from a homogeneous batch. The pronounced Brownian motion of the particles promotes efficient mixing and, consequently, a high degree of batch homogeneity. At the same time, for particles smaller than 10 nm, Brownian motion also increases the frequency of collisions both between particles and with the container walls, thereby promoting aggregation and wall deposition. The combined standard uncertainty was calculated as

42 $$u_{c}={\left(u_{bb}^{2}+u_{\mathrm{rep}}^{2}+u_{\mathrm{lts}}^{2}+u_{\mathrm{bias}}^{2}\right)}^{1/2}$$

Application of eq 42 yielded combined standard uncertainties, *u*_*c*_, of 3.9% for *D*, 6.6% for σ, 9.2% for *N*, and 8.3% for *c*. In all cases, these values were substantially lower than the corresponding target uncertainties of 5%, 10%, 20%, and 20%. The expanded uncertainty was calculated as *U* = *ku*_*c*_ using an expansion factor of *k* = 2. The resulting *U* values and the individual contributions are shown in Figure 6 and listed in Table 4. A summary of the metrological traceability of the measurands is provided in section 4 of the Supporting Information.

**Table 4:** Assigned Values, Individual Uncertainty Contributions, Combined Standard Uncertainties, and Expanded Uncertainties for the Measurands^a^

parameter	diameter, *D*	size distribution width, σ	number density, *N*	concn, *c*
unit	nm	nm	10^–6^ mol L^–1^	g L^–1^
assigned value	5.83	1.33	3.74	3.04
Uncertainty contributions				
homogeneity, *u*_*bb*_	0.03 (0.6%)	0.00 (0.0%)	0.05 (1.4%)	0.00 (0.0%)
repeatability, *u*_rep_	0.13 (2.2%)	0.05 (4.0%)	0.15 (3.9%)	0.04 (1.5%)
stability, *u*_lts_	0.14 (2.5%)	0.06 (4.6%)	0.21 (5.5%)	0.08 (2.7%)
bias, *u*_bias_	0.11 (1.9%)	0.03 (2.4%)	0.23 (6.1%)	0.24 (7.8%)
Combined standard uncertainty				
*u*_c_	0.22 (3.9%)	0.09 (6.6%)	0.34 (9.2%)	0.25 (8.3%)
Expanded uncertainty (*k* = 2)				
*U*	0.5 (9%)	0.18 (14%)	0.7 (19%)	0.5 (17%)

a Relative uncertainties are given in parentheses.

### Post-Certification Measurand Values

At the time of certification as reference material BAM-N008 in 2023,^24^ the particles had already undergone prolonged storage under sealed conditions since 2016, corresponding to a pre-certification aging period of approximately seven years. Such an extended history provides an inherently stringent baseline for assessing material stability. To further probe the robustness of the certified properties beyond this already considerable time scale, post-certification measurements were performed in 2026. The resulting mean values of the measurands, derived from six independent measurements on six randomly selected samples (*n* = 6), are *D* = (6.02 ± 0.01) nm, σ = (1.27 ± 0.04) nm, *N* = (3.57 ± 0.01) × 10^−6^ mol L^−1^, and *c* = (3.11 ± 0.02) g L^−1^. These data points are included as gray crosses in Figure 4, alongside the long-term stability data set. The post-certification measurements deviate from the extrapolated linear trends, indicating that the early drift underlying the linear model does not persist at a constant rate over longer time scales, consistent with transient equilibration of the particle–PAA system rather than open-ended aging. Notably, all post-certification values lie within the expanded uncertainties (*k* = 2) of the assigned values. The observed deviations from the certified reference values were consistently smaller than expected from the combined uncertainty budget. This indicates a high degree of temporal stability and suggests that aging effects remain negligible even over time scales longer than a decade. Accordingly, the originally assigned shelf life appears to have been highly conservative.

### Colloidal Stability Assessed by Dynamic Light Scattering (DLS)

The long-term stability of the silver nanoparticles is notable because their small size and correspondingly high diffusion coefficients would generally be expected to promote aggregation. To clarify the origin of this stability, we investigated the effect of the PAA concentration on the colloidal behavior. We aimed to use the minimum amount of stabilizer required to suppress aggregation while avoiding excessive surface modification that could alter the intrinsic particle properties.

Samples containing 3–4 wt % PAA (relative to the dry mass) initially yielded dispersions that appeared stable. However, after six months, SAXS showed a decrease in both particle number and particle diameter, with no evidence of aggregation. This behavior was attributed to the gradual deposition of nanoparticles on the walls of the storage containers. Because optically non-transparent brownish polyethylene bottles were used, this process was not immediately evident, although a visible nanoparticle film had formed after prolonged storage, as demonstrated in Figure S4. Increasing the PAA content to 13–14 wt % substantially improved colloidal stability, as the SAXS patterns remained unchanged after six months. These dispersions were sufficiently stable for catalytic studies on the reduction of 4-nitrophenol^17^ and for use as reference materials in an interlaboratory SAXS comparison.^15^ To further improve long-term stability, a PAA content of 30 wt % was ultimately selected (see Experimental Section), making stability over several years likely.

Steric stabilization is known to require a polymer layer above a critical thickness. According to Tadros, dispersions become unstable when the thickness of the adsorbed polymer layer falls below approximately 5 nm.^25^ As a rule of thumb, adsorption layer thicknesses in the range of 5–20 nm are typically required for long-term colloidal stability, depending on polymer chain length and solvent quality. Effective steric stabilization additionally requires that the adsorbed stabilizer chains are solvated by the continuous phase and retain sufficient conformational freedom, since chain compression upon particle approach must generate a repulsive osmotic and elastic contribution. The thickness of the stabilizing shell was estimated from the difference between the hydrodynamic diameter and the metallic core diameter. Here, the hydrodynamic diameter *D*_H_, determined by DLS, represents the total particle diameter, whereas the core diameter *D* is obtained from SAXS. Because the X-ray scattering contrast of the silver core (6.79 × 10^11^ cm^–2^) is much greater than that of the PAA shell (0.49 × 10^11^ cm^–2^) , the square of the scattering contrast of the core exceeds that of the shell by approximately a factor of 225. Owing to the much greater X-ray scattering contrast of the silver core relative to the PAA shell, together with the strongly reduced effective density of the solvated, conformationally free polymer shell compared to bulk PAA, the shell’s contribution to the total SAXS signal is negligible. This is further supported by the absence of any low-*q* feature in the measured SAXS patterns beyond that expected for the metallic core (Figure 2), consistent with treating the polymer shell as effectively invisible in SAXS.

Dynamic light scattering measurements were carried out for samples containing 3–4 wt %, 13–14 wt %, and 30 wt % PAA. The intensity autocorrelation functions, *G*_2_(τ) – 1, were analyzed by the method of cumulants as described by Frisken.^26^ Further details are provided in the Supporting Information. Representative *G*_2_(*τ*) – 1 functions and the corresponding fits are shown in Figure 7.

**Figure 7:**
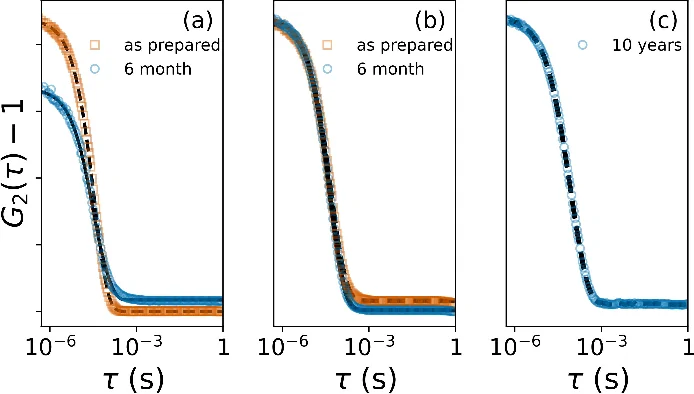
Dynamic light scattering (DLS) intensity autocorrelation functions, *G*_2_(τ) – 1, as a function of the delay time, τ, for nanoparticle dispersions with different poly­(acrylic acid) (PAA) contents and storage times. (a) Nanoparticles stabilized with 3–4 wt % PAA, measured in the as-prepared state and after 6 months of storage. (b) Nanoparticles stabilized with 13–14 wt % PAA, measured in the as-prepared state and after 6 months of storage. (c) Nanoparticles stabilized with 30 wt % PAA, measured after 10 years of storage. Symbols represent the experimental data, and the dashed black lines represent fits to the autocorrelation functions.

Within the first three months after synthesis, particles stabilized with 3–4 wt % PAA showed a hydrodynamic diameter of *D*_H_ = (11.6 ± 0.2) nm and a polydispersity index (PI) of 0.04 ± 0.02, indicating a highly monodisperse population.^27^ After six months of storage under ambient conditions, the hydrodynamic diameter decreased slightly to *D*_H_ = (10.4 ± 0.4) nm. No aggregation was observed in solution; however, the data quality deteriorated and the polydispersity index increased markedly to 0.37 ± 0.02. These findings suggest partial loss of particles due to deposition at the vial wall rather than aggregation. For samples containing 13–14 wt % PAA, the initial hydrodynamic diameter was *D*_H_ = (15.2 ± 0.4) nm and the PI was 0.13 ± 0.03, consistent with moderate polydispersity. No significant changes in either parameter were observed after six months, confirming good colloidal stability. By contrast, nanoparticles containing 30 wt % PAA showed a much larger hydrodynamic diameter of *D*_H_ = (51.4 ± 0.4) nm and a polydispersity index of 0.30 ± 0.10, indicating a broad size distribution. The thickness of the polymer shell, estimated as (*D*_H_ – *D*)/2, was approximately 2.9, 4.7, and 23 nm for the low-, intermediate-, and high-PAA samples, respectively. These values are consistent with the observed stability trends and underline the need for a minimum steric layer thickness to ensure long-term colloidal stability. A schematic illustration of the core–shell structure with scale-accurate proportions is shown in Figure S5.

In addition to the layer thickness, the surface coverage of the stabilizing polymer plays a crucial role. Typically, adsorption densities in the range of 0.5 to 5 mg m^–2^ of particle surface are required to achieve sufficient steric stabilization. Therefore, we examine the stabilizer mass per unit surface area. For a mass of 3.04 g L^–1^ of silver and a relative amount of 3–4, 13–14, and 30 wt % of stabilizer, the respective concentrations of poly­(acrylic acid) are 0.12 g L^–1^, 0.49 g L^–1^, and 1.30 g L^–1^. The total silver particle surface area, calculated from the core diameter and the particle number concentration, amounts to 240 m^2^ *L* ^–1^. The amounts of poly­(acrylic acid) are Γ = 0.53 mg m^–2^, 2.06 mg m^–2^, and 5.42 mg m^–2^. These are the maximum values of adsorbed polymer on the silver particle surfaces in the case that all poly­(acrylic acid) adheres to the silver nanoparticle surface. When having a look at the literature on non-covalently bound polymeric stabilizers, for example, polystyrene on silica, the adsorbed amounts are typically in the range Γ ≈ 1–5 mg m^–2^. With a broader range the amount is approximately 0.1–10 mg m^–2^ depending on the polymer, particle surface, and solvent conditions.^28^ In conclusion, it is not surprising that the concentration of 0.53 mg m^–2^ of poly­(acrylic acid) was insufficient to provide long-term stability. A concentration of 2.06 mg m^–2^ might have been sufficient, but 5.42 mg m^–2^ seems to be a reasonable choice for providing at least a decade of colloidal stability as we observe in this study. Determining the fraction of poly­(acrylic acid) adsorbed to the particle surface versus that present unbound in solution is of interest but lies beyond the scope of the present study, which focuses on the use of SAXS for nanoparticle characterization.

To summarize, achieving long-term stability comes at the cost of a significant increase in PAA content and polydispersity in hydrodynamic diameter.

### Colloidal Stability Assessed by X-ray Radiation

An important consideration in X-ray scattering experiments is whether ionizing radiation exposure may alter the sample during measurement. While the silver core is generally resistant to X-ray-induced changes, the poly­(acrylic acid) stabilizer is potentially more sensitive to radiation effects. Leveraging data from the interlaboratory study,^15^ we examined silver nanoparticles containing 3–4 wt % and 13–14 wt % stabilizer under synchrotron conditions. These measurements enabled a direct assessment of radiation tolerance in the dispersions, particularly regarding the polymer shell’s stability under intense X-ray irradiation. Our results revealed substantially faster aggregation for nanoparticles stabilized with 3–4 wt % PAA compared to those with 13–14 wt % PAA. The aggregation process followed diffusion-limited colloidal aggregation, yielding loosely packed mass-fractal aggregates with a fractal dimension of *d*_f_ = 1.7 (see Figures S6 and S7). For the 3–4 wt % PAA samples, aggregation began after approximately 1.5 s, while the 13–14 wt % PAA samples initiated aggregation only after 90 s. The corresponding radiation doses at aggregation onset were 4.65 × 10^4^ Gy and 2.79 × 10^6^ Gy, respectively (see Figure S8). No aggregation was observed for laboratory SAXS measurements, even after X-ray exposure times of up to 24 h. This discrepancy is explained by the dose rate for synchrotron SAXS, which was approximately 3.5 × 10^5^ times higher than that for laboratory SAXS. Further details are provided in the Supporting Information.

## Conclusions

This study shows that small-angle X-ray scattering (SAXS) is a robust and metrologically traceable method for certifying nanoparticle reference materials. The silver nanoparticle reference material BAM-N008 remained colloidally stable for at least ten years without special storage precautions. Homogeneity, stability, and uncertainty studies established traceable values for particle diameter and number density, as well as reliable reference values for size-distribution width and mass concentration. The certified measurand values and expanded uncertainties met or exceeded the target criteria, confirming the suitability of the material for analytical method validation and quality control. These results support further development in nanoparticle metrology and the practical use of certified reference materials. BAM-N008 is recognized as a certified SAXS reference material, with its assigned values for diameter, size-distribution width, number concentration, and mass concentration defined and traceable to the SAXS measurement model used here, specifically referencing the metallic silver core. It is not designed as a universal nanoparticle size reference independent of measurement technique and is not suitable for certifying hydrodynamic sizing methods like DLS, which measure the hydrated polymer shell rather than the metallic core.

## Supplementary Material



## Notes

Photograph and description of the reference-material vial, along with comparative SAXS measurements to address potential instrument-related biases are provided, as well as results on both short- and long-term stability, details of the approaches used to ensure metrological traceability and absolute calibration, and outlines of the experimental protocols and fitting procedures applied in DLS analyses. In addition, data and modeling related to radiation-induced aggregation, as well as supplementary schematic illustrations and quantitative figures are provided (PDF)
