# Supplementary material for: Nanoparticle Analysis for Reference Materials: The Small-Angle X‑ray Scattering Method
Source: Langmuir. 2026 Sep 23;42(39):29084–95. doi: 10.1021/acs.langmuir.6c03792 (PMC13648265; doi:10.1021/acs.langmuir.6c03792)
Supplement: Supplementary file 1 [file la6c03792_si_001.pdf]

Supporting Information

Nanoparticle Analysis for Reference Materials: The Small-Angle X-ray Scattering Method

Andreas F. Thünemann*

Bundesanstalt für Materialforschung und -prüfung (BAM), Unter den Eichen 87, 12205 Berlin, Germany

E-mail: andreas.thuenemann@bam.de

Contents

1 Reference Material Bottle **2**

2 Instrument-Related Bias **2**

3 Stability **3**

3.1 Short-Term Stability 3

3.2 Long-Term Stability: Study Design 5

3.3 Long-Term Stability: Statistical Analysis 6

4 Metrological Traceability **9**

5 Colloidal Stability Assessed by Dynamic Light Scattering (DLS) **11**

5.1 DLS Experiments 11

5.2 DLS Data Evaluation 11

6 Colloidal Stability Assessed by X-ray Radiation **12**

6.1 Experimental 14

6.2 X-ray Induced Aggregation 15

1 Reference Material Bottle

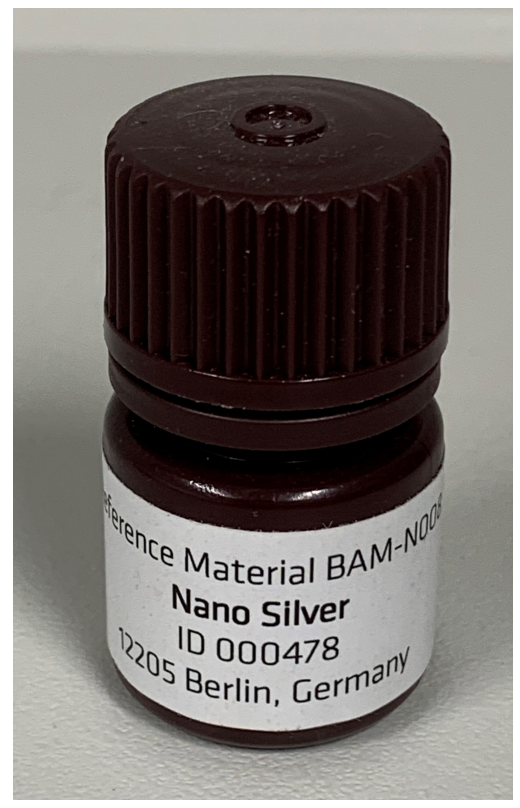

Figure S1: Bottle of the reference material BAM-N008 containing 5 mL of an aqueous dispersion of silver nanoparticles.

2 Instrument-Related Bias

The homogeneity, short-term stability, and long-term stability studies were carried out using the SAXS instrument described in the Experimental Section of the main text. Instrument 1 is a line-focus (slit-focus) Kratky-type system introduced in its present form in 2000.[1] Owing to its short sample-to-detector distance, it is particularly well-suited for measuring nanoparticles in solution. In combination with the line-focus geometry, this configuration enables precise measurement of the scattering intensity of water as a secondary standard for absolute intensity calibration.[2] Accurate absolute intensity measurements are essential for reliable determination of particle number and mass concentrations. Instrument 1 was also used in an interlaboratory comparison for determining the size distribution of silver nanoparticles.[3] These characteristics demonstrate its suitability for developing a nanoparticle reference material. Nevertheless, potential instrument-related bias must be excluded. To assess potential instrument-related bias, the silver nanomaterial was additionally measured using two SAXS instruments with substantially different designs. Instrument 2 is a commercial pinhole laboratory system operated with a vertical X-ray beam and has been demonstrated to provide

robust and traceable measurements of particle number concentration for gold nanoparticles.[4]

Instrument 3 is a non-commercial synchrotron-radiation instrument routinely used for metrological studies.[5,6] Measurements with instrument 2 were performed in accordance with the manufacturer's protocol, whereas measurements with instrument 3 followed the procedure established by PTB.[7] Ten randomly selected samples were measured with instrument 2, and the data were evaluated in the same manner as for instrument 1. The overall mean values obtained with instrument 2 were $D = (5.94 \pm 0.16)\,\mathrm{nm}$, $\sigma = (1.32 \pm 0.05)\,\mathrm{nm}$, $N = (3.85 \pm 0.28) \times 10^{-6}\,\mathrm{mol\,L^{-1}}$, and $c = (3.27 \pm 0.07)\,\mathrm{g\,L^{-1}}$.

Two randomly selected samples were measured with instrument 3 at PTB. Data analysis yielded D values of 6.25 and 5.86 nm and σ values of 1.37 and 1.28 nm. In this case, N and c could not be determined, as the scattering intensities were not available on an absolute scale. The results obtained with instruments 2 and 3 agree with those from the homogeneity study performed with instrument 1. These findings indicate that the measurement results are not significantly influenced by the specific SAXS instrument used. This conclusion is consistent with the previously reported interlaboratory comparison on silver nanoparticles.[3]

3 Stability

3.1 Short-Term Stability

The robustness of the material toward external influences was evaluated using five randomly selected samples. In particular, the effects of temperature variation and transportation were investigated. To assess temperature stability, one sample was stored at −40 °C, a second at 4 °C, and a third at 70 °C, each for 7 days. The remaining two samples were subjected to transportation stress by being carried as luggage on two intercontinental flights, each lasting 10 h. After temperature treatment or transport, all five samples were analyzed by SAXS.

An overview of the results is shown in Figure S3 (black circles). For comparison, the mean values obtained in the homogeneity study are also included (red squares), along with

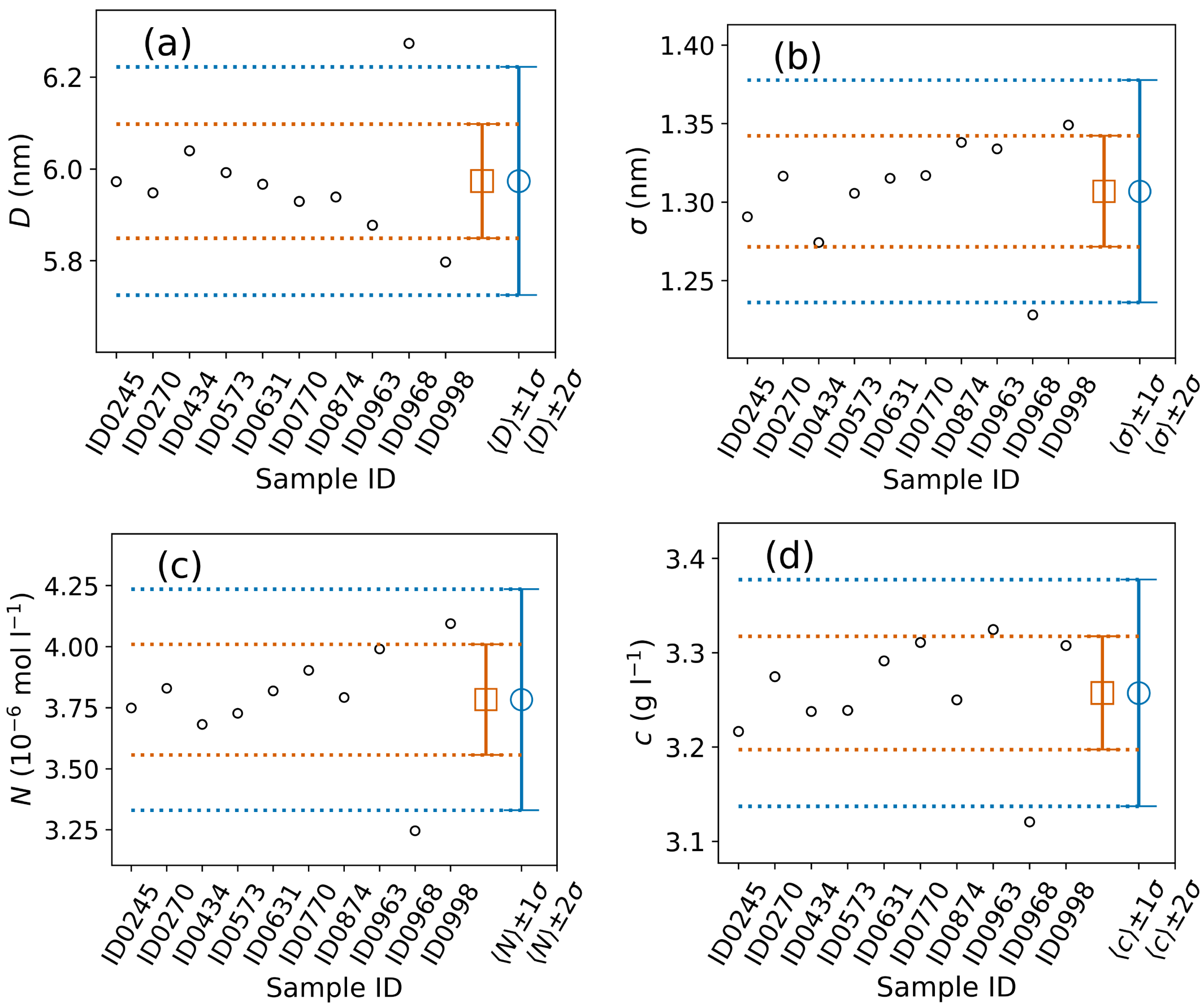

Figure S2: Results from measurements with instrument 2: (a) mean particle diameter D; (b) size distribution width σ; (c) number concentration N; (d) mass concentration c. For each panel, black open circles denote individual samples (ID0245–ID0998). The orange square and the blue circle mark the overall mean of all samples, with error bars indicating ± 1 and ± 2 standard deviations, respectively; the corresponding intervals are indicated by dotted orange and blue lines.

error bars corresponding to 1 standard deviation (red) and 2 standard deviations (blue). One value each for D, σ, and N, as well as two values for c, fell outside the interval defined by ± 2 standard deviations of the homogeneity study (horizontal dotted blue lines). This observation indicates that the applied external influences can induce small but detectable changes in the material properties. To minimize the risk of particle changes, exposure to temperatures outside $(20 \pm 3)\,^{\circ}$C should be avoided, and transportation times should be kept as short as practicable.

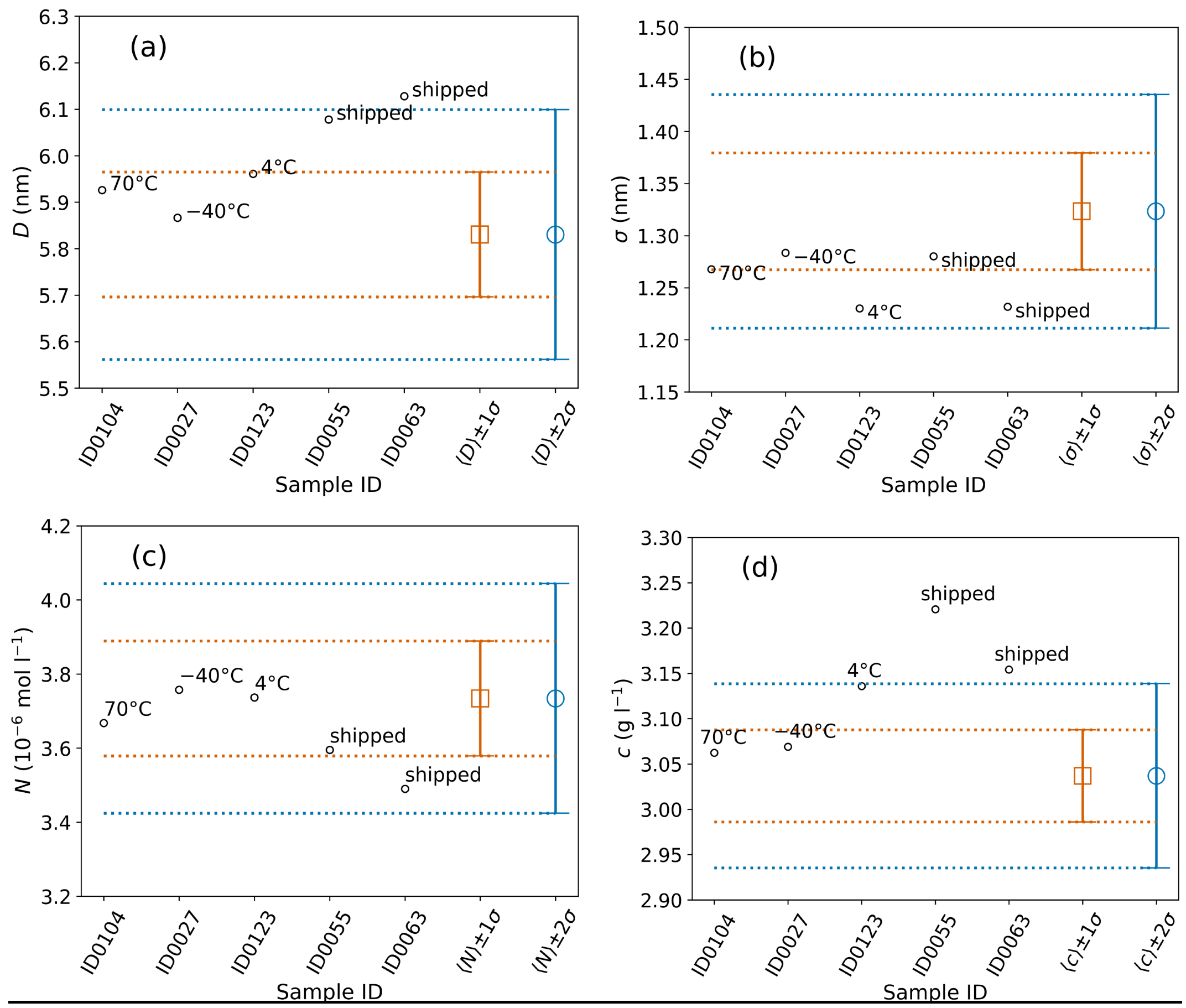

Figure S3: Results of the short-term stability study showing (a) particle diameter (D), (b) size distribution width (σ), (c) particle number density (N), and (d) mass concentration (c). Black circles represent individual measurements, with test conditions indicated adjacent to each data point. Red squares denote mean values obtained from the homogeneity study; red and blue error bars correspond to ± 1 and ± 2 standard deviations, respectively.

3.2 Long-Term Stability: Study Design

A long-term stability study was initiated after it had been verified that the changes in the measurands caused by temperature variation and transport were substantially smaller than the target uncertainties (see section 3.1). The start of the study ($t = 0$) was defined as the first day of the homogeneity study, which was conducted on days 354, 355, and 356 after bottling. The comparatively long interval between bottling and the start of the homogeneity study was chosen to assess whether colloidal stability was sufficiently high to support the development of a reference material.

The long-term stability study was conducted over 4 years using 30 randomly selected bottles stored under the specified shelf-life conditions, i.e., in the dark at an ambient temperature of $(20 \pm 3)\,^\circ$C. For each measurement occasion, one of these bottles was selected at random. The measurements were performed whenever instrument time was available rather than according to a statistically optimized sampling schedule.

3.3 Long-Term Stability: Statistical Analysis

In the univariate linear model described in Clause B.3 of ISO 33405:2024,[8] the individual values of a measurand, y_i, are related to the corresponding measurement times, x_i, according to

$$y_i = b_0 + b_1 x_i \tag{1}$$

where b_0 and b_1 denote the estimated intercept and slope, respectively. The regression coefficients were calculated as

$$b_1 = \frac{\sum_{i=1}^{n}(x_i - \bar{x})(y_i - \bar{y})}{\sum_{i=1}^{n}(x_i - \bar{x})^2} \tag{2}$$

and

$$b_0 = \bar{y} - b_1 \bar{x} \tag{3}$$

where $\bar{x}$ and $\bar{y}$ are the means of the corresponding observations. The standard errors of b_1 and b_0 were calculated as

$$s(b_1) = s\left(\sum_{i=1}^{n}(x_i - \bar{x})^2\right)^{-1/2} \tag{4}$$

where

$$s^2 = \frac{1}{n-2}\sum_{i=1}^{n}(y_i - b_0 - b_1 x_i)^2 \tag{5}$$

and

$$s(b_0) = s(b_1)\left(\frac{1}{n}\sum_{i=1}^{n} x_i^2\right)^{1/2} \tag{6}$$

The fitted values of b_0 and b_1 from eqs 2 and 3 were substituted into eq 1, and the resulting regression lines are shown in Figure 4 of the main text (black solid lines) over a 10-year period. The slope was negative for D and positive for σ, N, and c, indicating a decrease in D and increases in σ, N, and c over time.

The statistical significance of these trends was evaluated according to Clause B.3.4 of ISO 33405:2024,[8] which describes a t test for assessing whether b_1 differs significantly from zero. The corresponding t statistic is given by

$$t_{b_1} = \frac{|b_1|}{s(b_1)} \tag{7}$$

and was compared with the two-tailed critical value $t_{95,n-2}$ of Student's t distribution for $n-2$ degrees of freedom at the 95% confidence level. For $n = 78$, $t_{95,n-2} = 1.99$. The resulting t_{b_1} values were 1.15 for D, 5.86 for σ, 3.50 for N, and 12.16 for c.

Because the value obtained for D was smaller than $t_{95,n-2}$, no statistically significant time-dependent change in particle diameter was detected. In contrast, the values obtained for σ, N, and c exceeded $t_{95,n-2}$, indicating statistically significant trends in these measurands.

Accordingly, changes in the measurand values over time must be accounted for. The fitted values of b_0 and b_1 were therefore used to estimate the response $\hat{y}$ at a given time $\hat{x}$ according to

$$\hat{y} = b_0 + b_1\hat{x} \tag{8}$$

The corresponding two-sided confidence interval at the 95% confidence level is given by

$$\hat{y}_{\pm} = \hat{y} \pm t_{95,n-2}\, s \left(\frac{1}{n} + \frac{(\hat{x}-\bar{x})^2}{\sum_{i=1}^{n}(x_i-\bar{x})^2} \right)^{1/2} \tag{9}$$

The regression lines calculated according to eq 8 and the corresponding confidence intervals derived from eq 9 are shown in Figure 4 of the main text as black solid and dotted lines, respectively.

If a linear trend is adopted as a tentative model for the observed increase in the measurand values, extrapolation can be used to estimate the storage lifetime (shelf life). Alternatively, interpolation can be used to define an initial monitoring time point at which the validity of the assigned measurand values should be reassessed.

The acceptable range for each measurand of BAM-N008 may be defined by an upper specification limit, L_{upr}, and a lower specification limit, L_{lwr}. Assuming a simple linear degradation trend, the confidence interval for a predicted future measurand value is given by eqs 8 and 9, where $\hat{x}$ denotes the time elapsed since the beginning of the stability study. The required shelf life is determined by setting eq 9 equal to each specification limit and solving for $\hat{x}$. The shortest positive solution defines the shelf life, t_{shelf}, according to

$$t_{\mathrm{shelf}} = \min\left(t_{\mathrm{s,upr}},\ t_{\mathrm{s,lwr}}\right) \tag{10}$$

where $t_{\mathrm{s,upr}}$ and $t_{\mathrm{s,lwr}}$ are the smallest positive solutions of

$$L_{\mathrm{upr}} = b_0 + b_1 t_{\mathrm{s,upr}} \pm t_{95,n-2}\, s \sqrt{\frac{1}{n} + \frac{(t_{\mathrm{s,upr}} - \bar{x})^2}{\sum_{i=1}^{n}(x_i - \bar{x})^2}} \tag{11}$$

$$L_{\mathrm{lwr}} = b_0 + b_1 t_{\mathrm{s,lwr}} \pm t_{95,n-2}\, s \sqrt{\frac{1}{n} + \frac{(t_{\mathrm{s,lwr}} - \bar{x})^2}{\sum_{i=1}^{n}(x_i - \bar{x})^2}} \tag{12}$$

Equations 11 and 12 yield four solutions in total. For convenience, the sum of squares is defined as

$$SS \equiv \sum_{i=1}^{n}(x_i - \bar{x})^2 \tag{13}$$

One solution of eq 11 is

$$t_{\mathrm{s,upr}} = -\frac{b_1(b_0 - L_{\mathrm{upr}})\, SS + s^2 t_{95,n-2}^2\, \bar{x} + s\, t_{95,n-2}\sqrt{\frac{1}{n} SS\left[b_1^2 SS - s^2 t_{95,n-2}^2 + n(b_0 - L_{\mathrm{upr}} + b_1\bar{x})^2\right]}}{b_1^2 SS - s^2 t_{95,n-2}^2} \tag{14}$$

and a second solution is

$$t_{\mathrm{s,upr}} = \frac{b_1(L_{\mathrm{upr}} - b_0)\,SS - s^2 t_{95,n-2}^2\,\bar{x} + s\,t_{95,n-2}\sqrt{\frac{1}{n}SS\left[b_1^2 SS - s^2 t_{95,n-2}^2 + n(b_0 - L_{\mathrm{upr}} + b_1\bar{x})^2\right]}}{b_1^2 SS - s^2 t_{95,n-2}^2} \tag{15}$$

The corresponding solutions for $t_{\mathrm{s,lwr}}$ are obtained by replacing L_{upr} with L_{lwr} in eqs 14 and 15. In the present study, solution 1 yielded estimates of $t_{\mathrm{s,upr}}$ of 10.85 years for D, 6.07 years for σ, 12.00 years for N, and 14.96 years for c. A summary of the regression parameters obtained from the long-term stability study is given in Table 2 of the main text.

The same principle used to estimate the shelf life may also be applied to define an initial monitoring time point for post-certification stability monitoring. To establish appropriate monitoring intervals, the observed rates of change of the measurands were considered. Assuming a linear trend, the estimated annual changes were $b_1 = -0.01\,\mathrm{nm\,year^{-1}}$ for D, $0.02\,\mathrm{nm\,year^{-1}}$ for σ, $0.04 \times 10^{-6}\,\mathrm{mol\,L^{-1}\,year^{-1}}$ for N, and $0.04\,\mathrm{g\,L^{-1}\,year^{-1}}$ for c. These results indicate that the four measurands differ clearly in their temporal behavior. Elucidation of the physicochemical origin of these differences would be of considerable interest, but such investigations are beyond the scope of the present work. At present, it is sufficient to note that the annual changes remain acceptable with respect to the predefined target uncertainties. To monitor any future variation in the measurand values, one monitoring point per year was selected.

4 Metrological Traceability

The certified values of the diameter and number density (number concentration) are based on the SAXS methods described in ISO 17867 and ISO 23484, respectively. The diameter and number density are traceable to the SI through calibrated measurements of length and amount of substance per volume. Details of the traceable determination of particle diameter are given in ISO 17867 and the references cited therein.[6,9] Details of the traceable

determination of particle number density are provided in ISO 23484 and the reference cited therein.[7]

Briefly, according to ISO 23484,[10] the differential scattering cross section of a sample is given by

$$\Delta\Sigma(q) = \frac{I(q)}{\Phi_0 T \Delta\Omega\, t_{\mathrm{exp}}\, \eta_{\mathrm{OE}}\, w}. \tag{16}$$

$\Delta\Sigma(q)$ represents the macroscopic differential scattering cross section per unit sample volume, $(1/V)(\mathrm{d}\sigma/\mathrm{d}\Omega)$. Following common convention in the SAXS literature, this quantity is expressed in units of reciprocal length (m^{-1}), the steradian being dimensionless in SI.[11] $I(q)$ is the measured scattering intensity, Φ_0 is the incident photon flux, T is the transmission, $\Delta\Omega$ is the solid angle, t_{exp} is the exposure time, η_{OE} is the detector quantum efficiency, and w is the sample thickness.

For water, the differential scattering cross section is

$$\Delta\Sigma_{\mathrm{H_2O}}(q) = \frac{I_{\mathrm{H_2O}}(q)}{\Phi_0 T \Delta\Omega\, t_{\mathrm{exp}}\, \eta_{\mathrm{OE}}\, w}, \tag{17}$$

where $I_{\mathrm{H_2O}}(q)$ is the measured scattering intensity of water. The scattering cross section of water arises from thermally driven density fluctuations related to its osmotic compressibility.[12] The purity of the water with respect to dissolved ions and gases is irrelevant to its use as a SAXS calibration standard, because the scattering cross section arises from bulk density fluctuations of the solvent rather than from discrete dissolved species.

Because $I(q)$ and $I_{\mathrm{H_2O}}(q)$ were measured consecutively under identical experimental conditions, that is, using the same SAXS instrument, the same capillary, and the same measurement time, the quantities Φ_0, T, $\Delta\Omega$, t_{exp}, η_{OE}, and w were identical for both measurements. Combining eqs 16 and 17 yields

$$\Delta\Sigma(q) = \frac{\Delta\Sigma_{\mathrm{H_2O}}(q)}{I_{\mathrm{H_2O}}(q)}\, I(q). \tag{18}$$

Here, $\Delta\Sigma_{H_2O}(q)$ is a tabulated constant[2] with a value of $1.65\,m^{-1}$ ($0.0165\,cm^{-1}$), and $I_{H_2O}(q)$ is constant in the range $0.5 < q < 2\,nm^{-1}$. Determination of $\Delta\Sigma(q)$ according to eq 18, including uncertainty propagation, was performed using the SAXS data reduction software SAXSquant (Anton Paar, Graz, Austria). A certified glassy carbon reference material was employed to verify the results obtained using water as an intensity calibration standard.[13] Traceability of the scattering vector q was established following the approach described by Smales and Pauw.[14] The accuracy of the q values was further cross-checked using silver behenate as a calibration standard.[15]

5 Colloidal Stability Assessed by Dynamic Light Scattering (DLS)

5.1 DLS Experiments

The DLS measurements were performed using a multi-angle ALV instrument (ALV-7004, ALV-GmbH, Langen, Germany) equipped with a He–Ne laser ($\lambda_0 = 632.8\,nm$). The scattering data were collected at $(23 \pm 1)\,°C$ over a scattering-angle range of $\theta = 26°$ to $146°$, using increments of 8°. In total, six measurements were carried out, with each angle measured for 60 s. Prior to measurement, the samples were diluted 1:100 with sodium hydroxide solution at pH 10 and filtered through an LCR filter (Millex-LCR, Merck, $0.45\,\mu m$ pore size). Since no significant angle dependence of the hydrodynamic radii could be detected,[16] the data measured at a scattering angle of 90° were selected for data evaluation.

5.2 DLS Data Evaluation

The intensity autocorrelation function is modeled by

$$G_2(\tau) - 1 = b + \beta \exp(-2\bar{\Gamma}\tau) \left(1 + \frac{\mu_2}{2}\tau^2\right)^2 \tag{19}$$

as introduced by Frisken[17] as a variant of Koppel's analysis of cumulants.[18] Here, b is a baseline, β is a scaling parameter, $\bar{\Gamma} = q^2\bar{D}$ is the mean relaxation frequency, and μ_2 is the second moment about the mean of the distribution of relaxation frequencies, $\mu_2 = \int_0^\infty G(\Gamma)\,(\Gamma - \bar{\Gamma})^2\,\mathrm{d}\Gamma$. The Stokes–Einstein relation provides the hydrodynamic diameter

$$D_\mathrm{H} = \frac{k_\mathrm{B}T}{3\pi\eta\bar{D}} \tag{20}$$

Inserting $\bar{D} = \bar{\Gamma}/q^2$ provides

$$D_\mathrm{H} = \frac{k_\mathrm{B}Tq^2}{3\pi\eta\bar{\Gamma}}, \tag{21}$$

where k_B is the Boltzmann constant, T is the temperature, and η is the viscosity of the solvent. The magnitude of the scattering vector is $q = (4\pi n/\lambda_0)\sin(\theta/2)$, where λ_0 is the wavelength of the light source in vacuum, n is the refractive index of the medium, and θ is the scattering angle. The polydispersity index is defined as $\mathrm{PI} = \mu_2/\bar{\Gamma}^2$, where PI values of ≤ 0.05 are considered as monodisperse.[19] A viscosity equal to that of water at 23 °C was assumed in eq 21 because no visually discernible increase in viscosity relative to water was observed for the diluted dispersions, consistent with the comparatively low molar mass of the poly(acrylic acid) stabilizer ($M_\mathrm{w} = 1800\,\mathrm{g\,mol^{-1}}$). The absence of angular dependence of the relaxation rate was previously confirmed for nanoparticles of the same synthesis and stabilization protocol (see Figure 3 in Kästner and Thünemann[16]), and was therefore not re-verified here.

6 Colloidal Stability Assessed by X-ray Radiation

We wanted to assess whether the nanoparticles are sensitive to X-ray radiation and utilized a laboratory and a synchrotron X-ray source. The high X-ray doses available at synchrotron sources may cause substantial radiation damage to nanoparticles during data collection, as has been clearly demonstrated for proteins[20] and other biological macromolecules.[21,22]

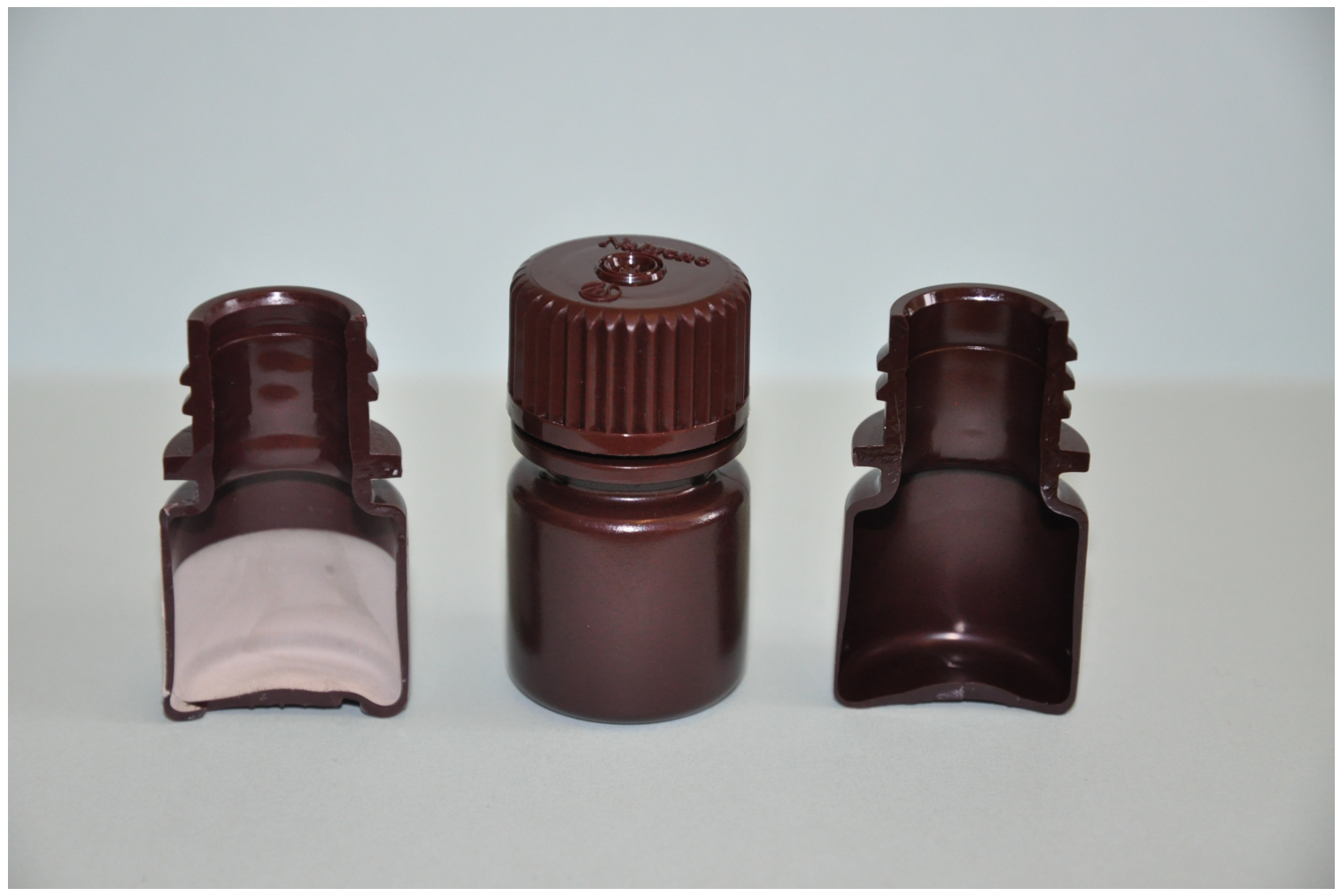

Figure S4: Intact and longitudinally sectioned amber bottles containing silver nanoparticle dispersions stabilized with 3–4 wt % and 13–14 wt % PAA after 6 months of storage under ambient conditions. Silver nanoparticle deposition on the vial wall is observed only for the dispersion containing 3–4 wt % PAA. For more details, see ref 16.

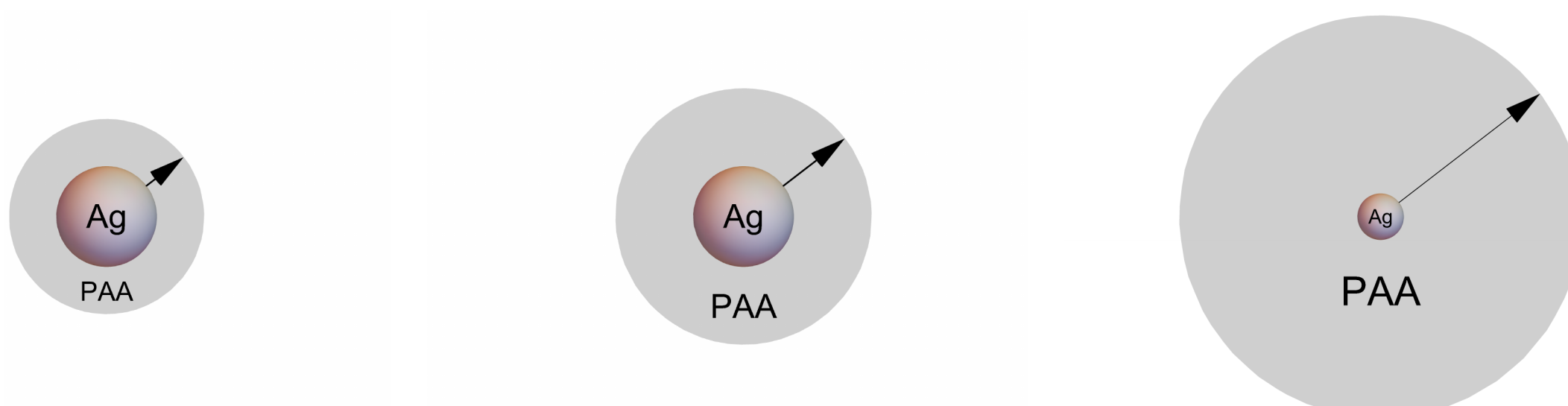

Figure S5: Schematic illustration of Ag@PAA core–shell particles with progressively increasing PAA shell thickness for PAA contents of 3–4, 13–14, and 30 wt % (from left to right). The Ag core is shown at the center, and the surrounding gray region denotes the PAA shell.

Typically, X-ray-induced damage leads to aggregation in dispersed samples.[22] For example, radiation damage in synchrotron SAXS studies of proteins has been investigated and discussed in detail by Hopkins and Thorne.[23] They found that the radiation sensitivity of the proteins studied varied by as much as six orders of magnitude. The radiation stability must be determined experimentally for each individual system.[22] With increasing beam power, more specialized experimental approaches become necessary, such as continuous-flow cells or dedicated injectors for “diffract-and-destroy” experiments.[24,25] In contrast to synchrotrons and X-ray free-electron lasers, the comparatively low flux of laboratory X-ray

sources usually does not cause measurable sample damage during exposure times required to obtain sufficient scattering signal for analysis. Inorganic nanoparticles generally exhibit greater radiation stability than organic nanoparticles because they are less susceptible to radical-mediated degradation. Recently, quantitative methods for assessing radiation damage in SAXS experiments have been introduced.[26] To achieve colloidal stability, the silver nanoparticles are here stabilized by PAA as organic ligands, which may be substantially more sensitive to radiation damage than the metallic core. Therefore, the sample with 3–4 wt % PAA is expected to be more prone to radiation damage than the sample with 13–14 wt % PAA.

6.1 Experimental

SAXS Measurements. Synchrotron SAXS measurements were carried out at beamline I22 of Diamond Light Source using an X-ray energy of 12.4 keV, corresponding to a wavelength of $\lambda = 0.100\,\mathrm{nm}$, and a sample-to-detector distance of 5.76 m. The magnitude of the scattering vector was defined as $q = (4\pi/\lambda)\sin\theta$. The nanoparticles were measured as prepared in aqueous dispersion at a concentration of $3\,\mathrm{g\,L^{-1}}$ and a temperature of $(23 \pm 2)\,°\mathrm{C}$.

Radiation damage is generally governed by the dose absorbed by the sample,[23] that is, the X-ray energy absorbed per unit mass, expressed in gray (Gy), where $1\,\mathrm{Gy} = 1\,\mathrm{J\,kg^{-1}}$. The absorbed dose was calculated as[23]

$$\mathrm{Dose} = \frac{fAE}{\rho l}\,t \tag{22}$$

where f is the flux density, A is the fraction of absorbed energy, E is the X-ray energy per photon, ρ is the sample density, l is the X-ray path length through the sample, and t is the exposure time.[27]

The photon flux density of the synchrotron X-ray beam at the sample position was estimated as $f = 7.0\times10^{19}\,\mathrm{photons\,s^{-1}\,m^{-2}}$ for a beam size of $0.1\times0.3\,\mathrm{mm^2}$. Using $A = 0.405$,

$E = 1.986 \times 10^{-15}\,\mathrm{J\,photon^{-1}}$, $\rho = 10^3\,\mathrm{kg\,m^{-3}}$, and $l = 1.8 \times 10^{-3}\,\mathrm{m}$, the resulting dose rate was estimated to be $3.1 \times 10^4\,\mathrm{J\,kg^{-1}\,s^{-1}}$. For comparison, a dose of $3.13 \times 10^5\,\mathrm{J\,kg^{-1}}$ would be required to heat the sample from 25 °C to its boiling point.

The laboratory instrument used was a line-focus system as described by Bergmann et al.,[1] operated with Cu K_α radiation at an energy of 8.041 keV and a wavelength of 0.15418 nm, using a tube voltage of 40 kV and a current of 40 mA. The photon flux density at the sample position was estimated as $f = 1.2 \times 10^{14}\,\mathrm{photons\,s^{-1}\,m^{-2}}$ for a beam size of $0.75 \times 0.2\,\mathrm{mm^2}$. Using $A = 0.57$, $E = 1.288 \times 10^{-15}\,\mathrm{J\,photon^{-1}}$, $\rho = 10^3\,\mathrm{kg\,m^{-3}}$, and $l = 1.0 \times 10^{-3}\,\mathrm{m}$, the corresponding dose rate was estimated to be $8.8 \times 10^{-2}\,\mathrm{J\,kg^{-1}\,s^{-1}}$.

The ratio of the dose rates for the synchrotron SAXS and laboratory SAXS measurements used here was therefore approximately 3.5×10^5.

The synchrotron SAXS data were reduced using the DAWN software package[28] and normalized with respect to acquisition time as well as the incident and transmitted X-ray flux before azimuthal integration. This procedure yielded one-dimensional scattering profiles over a scattering vector range of $0.1\,\mathrm{nm^{-1}} \leq q \leq 3\,\mathrm{nm^{-1}}$. The scattering contributions from the solvent (water) and the capillary were subtracted. The data were then placed on an absolute intensity scale ($\mathrm{m^{-1}}$) using the well-characterized scattering of glassy carbon.[13,29]

For the laboratory SAXS measurements, the data were calibrated to absolute intensity units using water, as described elsewhere,[2] with the aid of the SAXSquant software package (Anton Paar, Austria). The scattering patterns were recorded over the same scattering vector range as in the synchrotron SAXS measurements.

6.2 X-ray Induced Aggregation

We expected that the high photon flux available in synchrotron SAXS, corresponding to an estimated dose rate of $3.1 \times 10^4\,\mathrm{Gy\,s^{-1}}$, would induce aggregation. However, the timescale at which radiation-induced aggregation begins was initially unclear. A comparison of the scattering patterns of nanoparticles stabilized with 3–4 wt % and 13–14 wt % PAA measured

under synchrotron SAXS conditions is shown in Figure S6.

The left panel of Figure S6 shows the scattering curves recorded after synchrotron X-ray irradiation times of $t \approx 0$, 1, 2, 3, 4, and 5 s, corresponding to doses of 0, 3.1, 6.2, 9.3, 12.4, and 15.5×10^4 Gy. The arrow indicates an isoscattering point at $q \approx 0.46\,\mathrm{nm}^{-1}$ for the series of curves of the 3–4 wt % PAA nanoparticles. For these nanoparticles, irradiation times of $t \geq 2$ s, corresponding to doses of at least 6.2×10^4 Gy, lead to an increase in scattering intensity at q values below the isoscattering point. This behavior is consistent with the onset of aggregation.

In contrast, the scattering curves of the 13–14 wt % PAA nanoparticles overlap throughout the full irradiation series, as shown in the right panel of Figure S6. The time per frame was 10^{-2} s for the 3–4 wt % PAA and 1 s for the 13–14 wt % PAA nanoparticles. These values correspond to doses of 3.1×10^2 and 3.1×10^4 Gy per frame, respectively.

Figure S8 shows the evolution of χ^2_{red} as a function of irradiation time. Here, χ^2_{red} was used as a simple measure of the time-dependent changes in the scattering curves. It is defined as

$$\chi^2_{\mathrm{red}} = \frac{1}{N-M} \sum_{i=1}^{N} \left(\frac{I_{\mathrm{meas}}(q_i) - I_{\mathrm{ref}}(q_i)}{\sigma(q_i)} \right)^2 . \tag{23}$$

Using the first measured curve as the reference, $I_{\mathrm{ref}}(q)$, values of $\chi^2_{\mathrm{red}} \leq 1$ indicate the absence of statistically significant radiation-induced changes in the scattering data, whereas values of $\chi^2_{\mathrm{red}} > 1$ indicate significant changes. These two regimes are separated by a horizontal line in Figure S8. Data acquired in the regime $\chi^2_{\mathrm{red}} > 1$ should therefore be interpreted with caution. For 3–4 wt % PAA, this regime begins at $t \approx 1.5$ s, corresponding to a dose of 4.65×10^4 Gy. For 13–14 wt % PAA, the corresponding threshold is reached only after $t = 90$ s, corresponding to a dose of 2.79×10^6 Gy. From the difference in irradiation times at which χ^2_{red} exceeds unity, we conclude that 13–14 wt % PAA-stabilized nanoparticles remain stable in the synchrotron beam approximately 60 times longer than 3–4 wt % PAA-stabilized nanoparticles. In contrast to the synchrotron SAXS measurements, the scattering patterns of both 3–4 wt % and 13–14 wt % PAA-stabilized particles remained unchanged during irra-

diation with the X-ray beam of a laboratory SAXS instrument for 24 h. The total estimated dose over this period was 7.6×10^3 Gy. Because measurement times of 10–30 min are typically sufficient on the laboratory instrument to obtain data of reasonable quality,[16] we conclude that conventional laboratory X-ray sources do not induce significant radiation damage in either 3–4 wt % or 13–14 wt % PAA-stabilized nanoparticles.

Because radiation-induced aggregation is widely observed in nanoparticles and biomacromolecules, a more detailed analysis of the aggregation behavior of the 3–4 wt % PAA-stabilized particles is required. The presence of an isoscattering point in the curves displayed in Figure S6 suggests that the aggregation process can be described as a two-state transformation,[30] analogous to the occurrence of an isosbestic point in multicomponent spectra.[31] In addition, for irradiation times of $t \approx 5$ s, the low-q scattering intensity scales as $I(q) \sim q^{-1.75}$ below the isoscattering point, indicated by the black solid line in Figure S6. We therefore hypothesize that the two states correspond to (1) primary particles and (2) larger objects. In the absence of more detailed structural information, the larger objects are interpreted as fractal aggregates of the primary particles. Accordingly, the scattering curves were approximated as the sum of the scattering contributions from monomeric particles, $I_1(q)$, and fractal aggregates, $I_2(q)$:

$$I(q) = I_1(q) + I_2(q) \tag{24}$$

with

$$I_1(q) = N_1 \int_0^\infty \frac{1}{\sqrt{2\pi}\,\sigma R} \exp\left[-\frac{\ln^2(R/R_\mathrm{median})}{2\sigma^2}\right] I_\mathrm{s}(q, R, \Delta\eta)\,\mathrm{d}R \tag{25}$$

Here, N_1 denotes the number concentration of monomeric particles, R is the sphere radius, $\Delta\eta$ is the scattering length density difference between particle and matrix, σ is the width parameter of the size distribution, and R_median is the median radius. From these parameters, the mean radius was calculated as $R_\mathrm{mean} = R_\mathrm{median}\, e^{\sigma^2/2}$. The relative standard deviation of the size distribution was calculated as $\sigma_\mathrm{width}/R_\mathrm{mean} = \sqrt{e^{\sigma^2} - 1}$. The scattering contribution

of a single sphere is given by

$$I_{\mathrm{s}}(q,R,\Delta\eta) = \left[\frac{4}{3}\pi R^3 \Delta\eta \left(3\,\frac{\sin(qR) - qR\cos(qR)}{(qR)^3}\right)\right]^2. \tag{26}$$

The scattering contribution of the aggregates is described by

$$I_2(q) = N_2 \int_0^\infty \frac{1}{\sqrt{2\pi}\,\sigma R} \exp\left[-\frac{\ln^2(R/R_{\mathrm{median}})}{2\sigma^2}\right] I_{\mathrm{s}}(q,R,\Delta\eta)\,\mathrm{d}R \times S(q). \tag{27}$$

Here, N_2 is the number concentration of monomers incorporated in aggregates, and $S(q)$ is the interparticle fractal structure factor.[32,33] It is given by

$$S(q) = 1 + \frac{D_f}{r_0^{D_f}} \int_0^\infty r^{D_f - 3} e^{-r/\xi} \frac{\sin(qr)}{qr}\, r^2\,\mathrm{d}r, \tag{28}$$

which can be written in closed form as

$$S(q) = 1 + \left(\frac{\xi}{r_0}\right)^{D_f} \Gamma(D_f + 1)\, \frac{\sin[(D_f - 1)\tan^{-1}(q\xi)]}{(D_f - 1)\, q\xi\, [1 + (q\xi)^2]^{(D_f-1)/2}}. \tag{29}$$

The parameter D_f in eq 28 is the fractal dimension, and r_0 is the radius of the individual particles forming the fractal aggregate. Here, r_0 was interpreted as the sum of the primary-particle core radius and the shell thickness. A fixed value of $r_0 \approx 3\,\mathrm{nm}$ was used, implying that the particles are in close contact. The parameter ξ is a characteristic size of the fractal aggregate, more precisely, the cutoff length for the fractal correlation. It represents the distance above which the mass distribution within the aggregate is no longer described by the fractal scaling law. Here, an exponential cutoff function of the form $e^{-r/\xi}$ was used. To avoid ambiguous fitting results, ξ was fixed at an estimated value of approximately 64 nm, because the minimum accessible scattering vector, $q_{\mathrm{min}} = 0.1\,\mathrm{nm}^{-1}$, was too large to determine reliably how ξ evolves with irradiation time. Likewise, $R_{\mathrm{median}} \approx 2.87\,\mathrm{nm}$ and $\sigma \approx 0.21$ were kept constant, because the primary particles were assumed to remain

unchanged during aggregation. Accordingly, only the particle number concentrations N_1 and N_2 were treated as adjustable parameters. This approach yielded reasonable fits to the experimental scattering curves, as shown in Figure S7a.

The relative fraction of monomeric particles decreased from 100% at $t \approx t_0$ to 98% after 1 s, 82% after 2 s, 34% after 3 s, 17% after 4 s, and 9% after 5 s, as shown in Figure S7b. These results quantify the limited colloidal stability of the 3–4 wt % PAA-stabilized nanoparticles under exposure to synchrotron X-ray radiation. The fractal dimension of the aggregates was determined to be $D_f \approx 1.7 \pm 0.1$. Such a low fractal dimension is characteristic of loosely packed, open aggregate structures. Since $D_f < 2$, the aggregation of the 3–4 wt % PAA-stabilized nanoparticles can be assigned to diffusion-limited colloidal aggregation (DLCA).[34] Accordingly, the aggregates formed under these conditions are expected to exhibit loose mass-fractal morphologies. This aggregation behavior can be explained by degradation of the poly(acrylic acid) stabilizer under synchrotron X-ray irradiation, which prevents it from remaining adsorbed on the surfaces of the silver particles. As a consequence, the particles lose their steric stabilization and readily adhere upon collision during Brownian motion. This results in the formation of loosely packed mass-fractal aggregates.

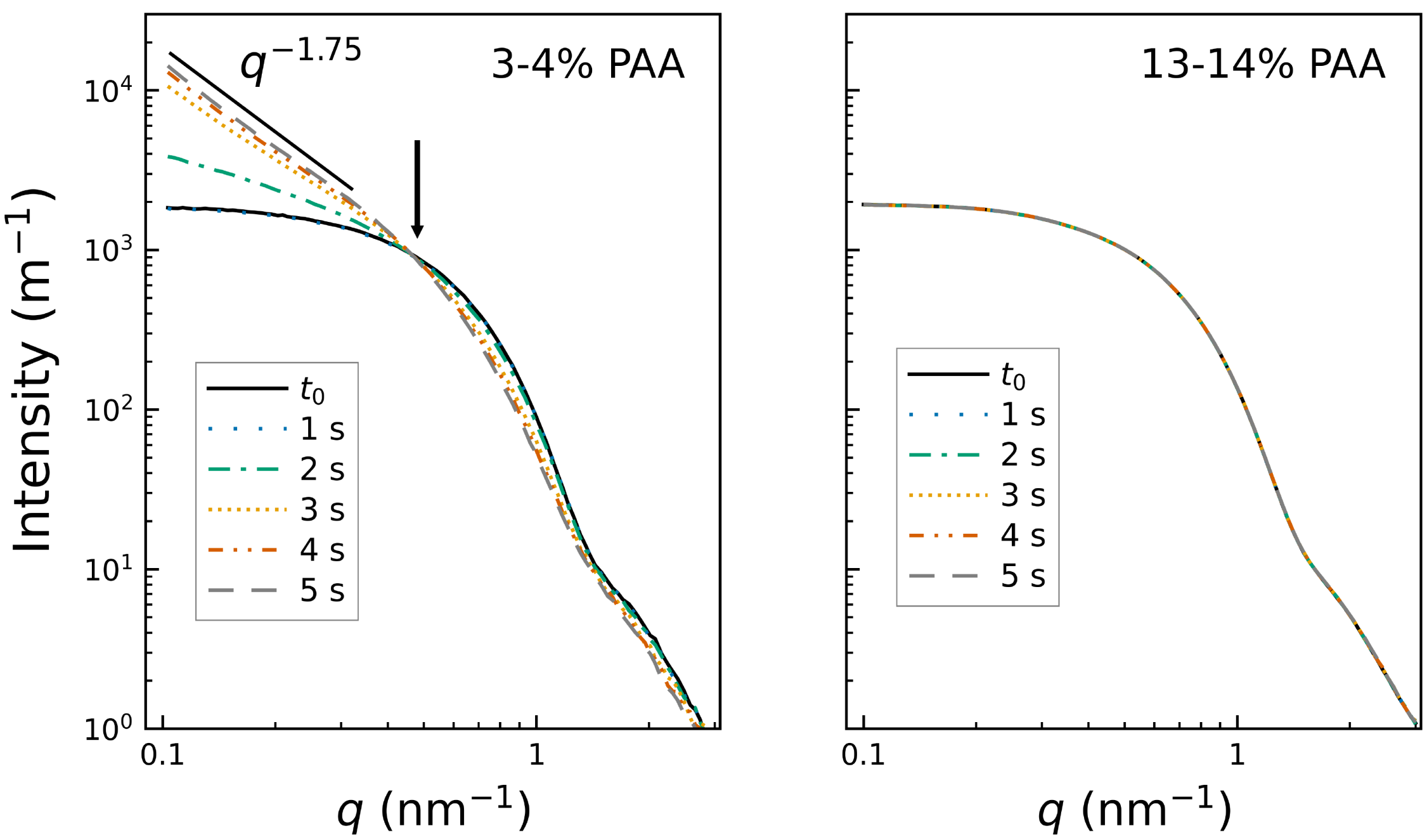

Figure S6: Scattering curves of nanoparticles stabilized with 3–4 wt % and 13–14 wt % PAA recorded after synchrotron X-ray irradiation times of $t \approx t_0$, 1, 2, 3, 4, and 5 s. The arrow in the left panel marks the isoscattering point at $q \approx 0.46\,\text{nm}^{-1}$ for the series of curves of the 3–4 wt % PAA particles. For these particles, curves recorded at $t \geq 2$ s exhibit an increase in scattering intensity at q values below the isoscattering point. For $t \approx 5$ s, the low-q scattering intensity follows a power-law dependence of $I(q) \sim q^{-1.75}$ below the isoscattering point, as indicated by the black solid line. In contrast, the scattering curves of the 13–14 wt % PAA particles remain superimposed throughout the irradiation series (right panel). For 3–4 wt % PAA-stabilized nanoparticles, frames were collected at 100 Hz using an acquisition time of 9 ms and a readout time of 1 ms. For 13–14 wt % PAA-stabilized nanoparticles, frames were collected at 1 Hz using an acquisition time of 100 ms and a readout/dead time of 900 ms. In both cases, the sample was continuously exposed to X-rays. For more details, see ref 16.

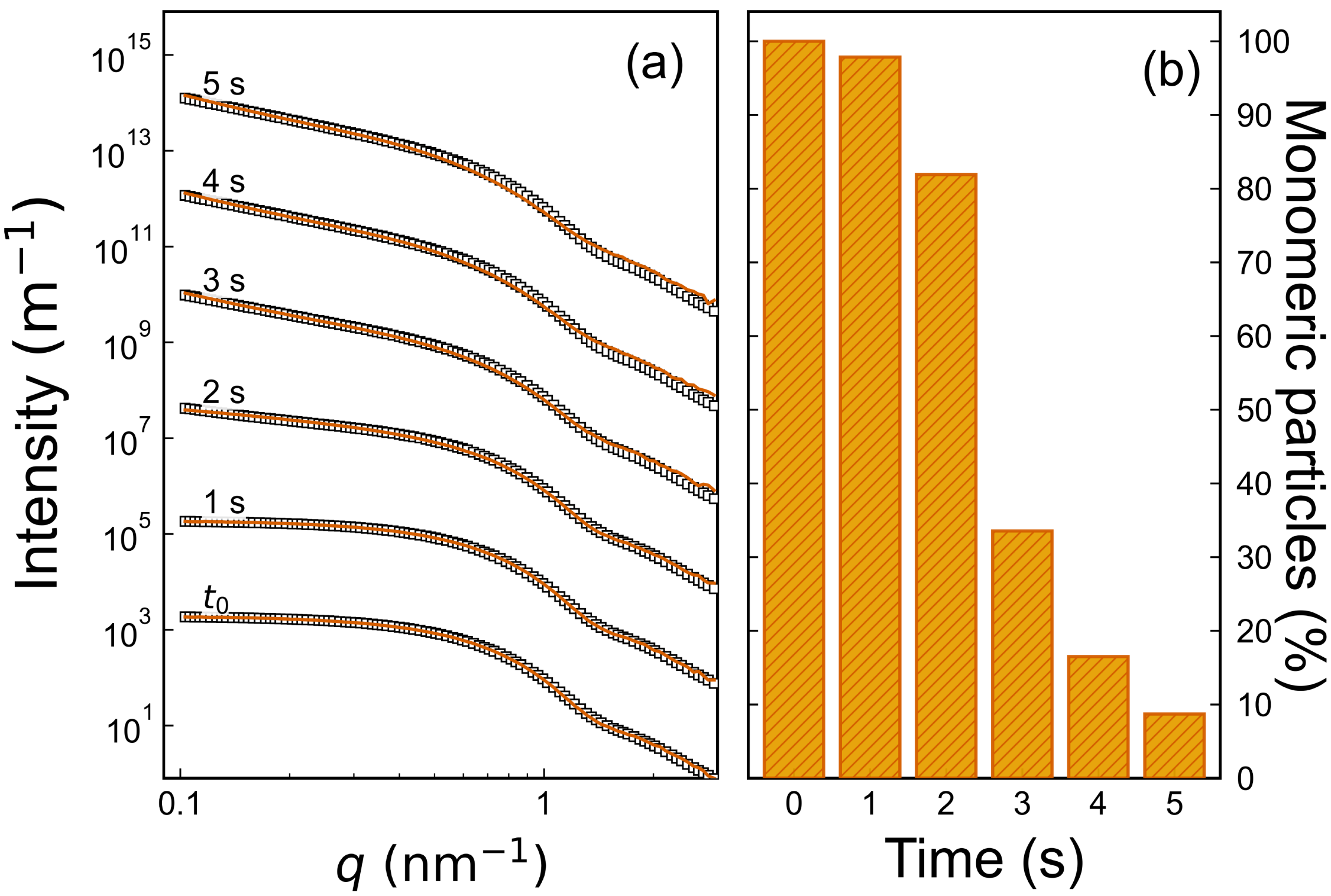

Figure S7: (a) Synchrotron SAXS data of the 3–4 wt % PAA-stabilized particles together with the corresponding curve fits obtained using a linear combination of monomeric particles and particle aggregates modeled as mass fractals with fractal dimension $D_f = 1.7 \pm 0.1$. Experimental data and fits are shown as symbols and orange solid lines, respectively. For clarity, the curves are vertically offset. (b) Relative amount of monomeric particles, N_1, as a function of irradiation time.

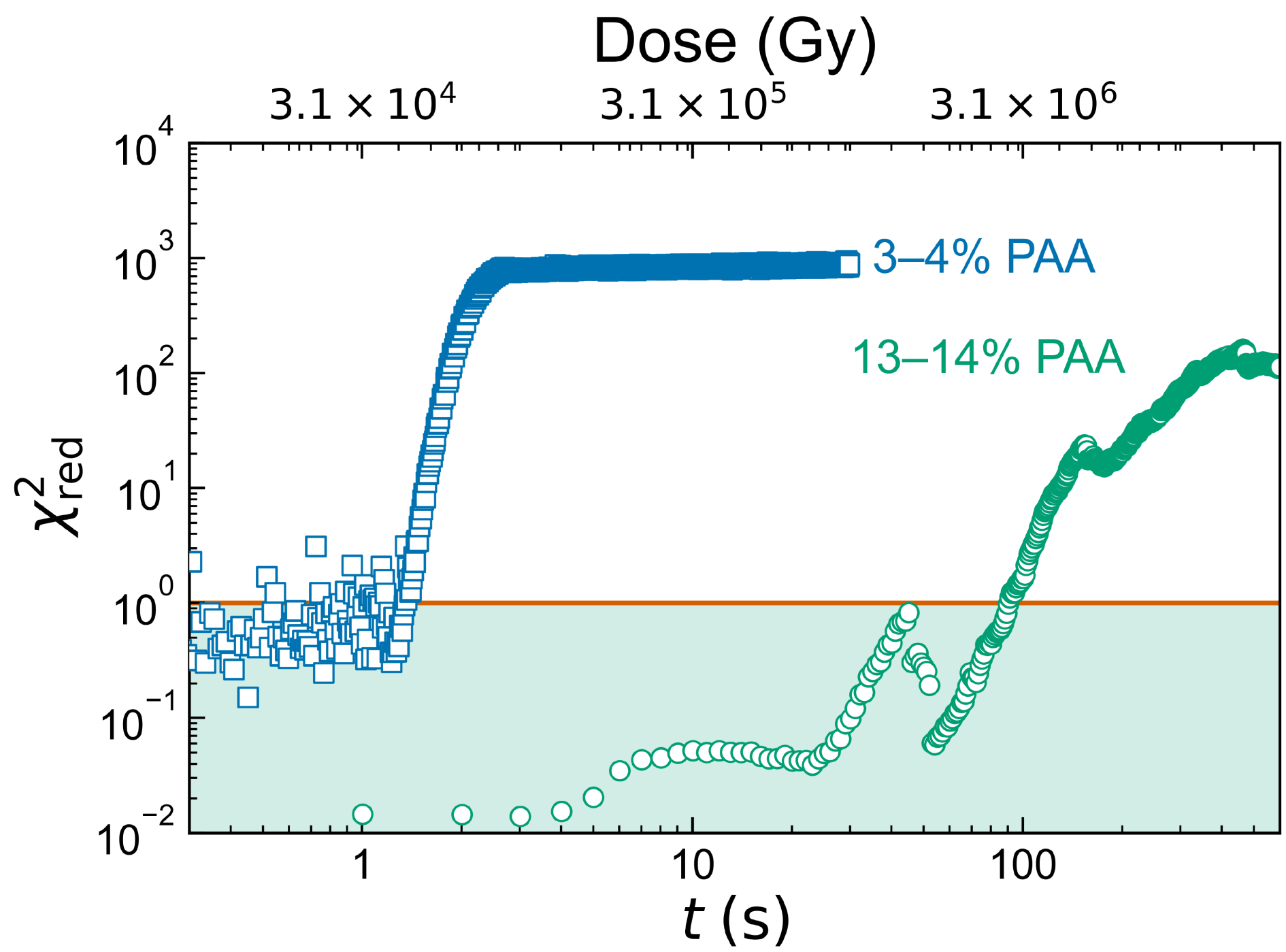

Figure S8: Comparison of the scattering data for nanoparticles stabilized with 3–4 wt % PAA and 13–14 wt % PAA, expressed in terms of χ^2_{red} as a function of irradiation time and corresponding absorbed X-ray dose. The quantity χ^2_{red} was used to quantify time-dependent changes in the scattering curves. Values of $\chi^2_{\text{red}} \leq 1$ indicate the absence of statistically significant changes in the data (green shaded region), whereas values of $\chi^2_{\text{red}} > 1$ indicate statistically significant changes. Accordingly, data acquired in the unshaded region above the horizontal line should be interpreted with caution. For the 3–4 wt % PAA sample, the transition to this region occurs at approximately $t \approx 1.5$ s, corresponding to an absorbed dose of 4.65×10^4 Gy. For the 13–14 wt % PAA sample, the corresponding threshold is reached only after $t = 90$ s, corresponding to an absorbed dose of 2.79×10^6 Gy.

References

(1) Bergmann, A.; Orthaber, D.; Scherf, G.; Glatter, O. Improvement of SAXS measurements on Kratky slit systems by Göbel mirrors and imaging-plate detectors. *J. Appl. Crystallogr.* **2000**, *33*, 869–875.

(2) Orthaber, D.; Bergmann, A.; Glatter, O. SAXS experiments on absolute scale with Kratky systems using water as a secondary standard. *J. Appl. Crystallogr.* **2000**, *33*, 218–225.

(3) Pauw, B. R.; Kästner, C.; Thünemann, A. F. Nanoparticle Size Distribution Quantification: Results of a Small-Angle X-ray Scattering Interlaboratory Comparison. *J. Appl. Crystallogr.* **2017**, *50*, 1280–1288.

(4) Xenocs SAS SAXS for Drug Development and Formulations. `https://www.xenocs.com/knowledge-base/saxs-for-drug-development-and-formulations/`, 2020; White paper, accessed 2026-06-04.

(5) Gleber, G.; Cibik, L.; Haas, S.; Höll, A.; Müller, P.; Krumrey, M. Traceable size determination of PMMA nanoparticles based on Small Angle X-ray Scattering (SAXS). *J. Phys.: Conf. Ser.* **2010**, *247*, 012027.

(6) Meli, F.; Klein, T.; Buhr, E.; Frase, C. G.; Gleber, G.; Krumrey, M.; Duta, A.; Duta, S.; Korpelainen, V.; Bellotti, R.; Picotto, G. B.; Boyd, R. D.; Cuenat, A. Traceable size determination of nanoparticles, a comparison among European metrology institutes. *Meas. Sci. Technol.* **2012**, *23*, 125005.

(7) Schavkan, A.; Gollwitzer, C.; Garcia-Diez, R.; Krumrey, M.; Minelli, C.; Bartczak, D.; Cuello-Nuñez, S.; Goenaga-Infante, H.; Rissler, J.; Sjöström, E.; Baur, G. B.; Vasilatou, K.; Shard, A. G. Number Concentration of Gold Nanoparticles in Suspension: SAXS and spICPMS as Traceable Methods Compared to Laboratory Methods. *Nanomaterials* **2019**, *9*, 502.

(8) International Organization for Standardization Reference materials – Approaches for characterization and assessment of homogeneity and stability. `https://www.iso.org/standard/84226.html`, 2024; ISO 33405:2024.

(9) Krumrey, M.; Gleber, G.; Scholze, F.; Wernecke, J. Synchrotron radiation-based x-ray reflection and scattering techniques for dimensional nanometrology. *Meas. Sci. Technol.* **2011**, *22*, 094032.

(10) International Organization for Standardization Determination of particle concentration – Small-angle X-ray scattering (SAXS). 2023; ISO 23484:2023.

(11) Glatter, O.; Kratky, O. *Small Angle X-ray Scattering*; Academic Press: London, 1982.

(12) Huang, C. et al. The inhomogeneous structure of water at ambient conditions. *Proc. Natl. Acad. Sci. U.S.A.* **2009**, *106*, 15214–15218.

(13) Allen, A. J.; Zhang, F.; Kline, R. J.; Guthrie, W. F.; Ilavsky, J. NIST Standard Reference Material 3600: Absolute Intensity Calibration Standard for Small-Angle X-ray Scattering. *J. Appl. Crystallogr.* **2017**, *50*, 462–474.

(14) Smales, G. J.; Pauw, B. R. The MOUSE project: a meticulous approach for obtaining traceable, wide-range X-ray scattering information. *J. Instrum.* **2021**, *16*, P06034.

(15) Huang, T. C.; Toraya, H.; Blanton, T. N.; Wu, Y. X-ray powder diffraction analysis of silver behenate, a possible low-angle diffraction standard. *J. Appl. Crystallogr.* **1993**, *26*, 180–184.

(16) Kästner, C.; Thünemann, A. F. Catalytic Reduction of 4-Nitrophenol Using Silver Nanoparticles with Adjustable Activity. *Langmuir* **2016**, *32*, 7383–7391.

(17) Frisken, B. J. Revisiting the Method of Cumulants for the Analysis of Dynamic Light-Scattering Data. *Appl. Opt.* **2001**, *40*, 4087–4091.

(18) Koppel, D. E. Analysis of Macromolecular Polydispersity in Intensity Correlation Spectroscopy: The Method of Cumulants. *J. Chem. Phys.* **1972**, *57*, 4814–4820.

(19) Roger, V.; Cottet, H.; Cipelletti, L. A New Robust Estimator of Polydispersity from Dynamic Light Scattering Data. *Anal. Chem.* **2016**, *88*, 2630–2636.

(20) Hong, X.; Hao, Q. Measurements of Accurate X-ray Scattering Data of Protein Solutions Using Small Stationary Sample Cells. *Rev. Sci. Instrum.* **2009**, *80*, 014303.

(21) Graewert, M. A.; Franke, D.; Jeffries, C. M.; Blanchet, C. E.; Ruskule, D.; Kuhle, K.; Flieger, A.; Schäfer, B.; Tartsch, B.; Meijers, R.; Svergun, D. I. Automated Pipeline for Purification, Biophysical and X-Ray Analysis of Biomacromolecular Solutions. *Sci. Rep.* **2015**, *5*, 10734.

(22) Jeffries, C. M.; Graewert, M. A.; Svergun, D. I.; Blanchet, C. E. Limiting Radiation Damage for High-Brilliance Biological Solution Scattering: Practical Experience at the EMBL P12 Beamline PETRAIII. *J. Synchrotron Radiat.* **2015**, *22*, 273–279.

(23) Hopkins, J. B.; Thorne, R. E. Quantifying Radiation Damage in Biomolecular Small-Angle X-ray Scattering. *J. Appl. Crystallogr.* **2016**, *49*, 880–890.

(24) van der Schot, G. et al. Open Data Set of Live Cyanobacterial Cells Imaged Using an X-ray Laser. *Sci. Data* **2016**, *3*, 160058.

(25) Kirby, N.; Cowieson, N.; Hawley, A. M.; Mudie, S. T.; McGillivray, D. J.; Kusel, M.; Samardzic-Boban, V.; Ryan, T. M. Improved Radiation Dose Efficiency in Solution SAXS Using a Sheath Flow Sample Environment. *Acta Crystallogr., Sect. D: Struct. Biol.* **2016**, *72*, 1254–1266.

(26) Brooks-Bartlett, J. C.; Batters, R. A.; Bury, C. S.; Lowe, E. D.; Ginn, H. M.; Round, A.; Garman, E. F. Development of Tools to Automate Quantitative Analysis of Radiation Damage in SAXS Experiments. *J. Synchrotron Radiat.* **2017**, *24*, 63–72.

(27) Kmetko, J.; Husseini, N. S.; Naides, M.; Kalinin, Y.; Thorne, R. E. Quantifying X-ray Radiation Damage in Protein Crystals at Cryogenic Temperatures. *Acta Crystallogr., Sect. D: Biol. Crystallogr.* **2006**, *62*, 1030–1038.

(28) Basham, M. et al. Data Analysis WorkbeNch (DAWN). *J. Synchrotron Radiat.* **2015**, *22*, 853–858.

(29) Zhang, F.; Ilavsky, J.; Long, G. G.; Quintana, J. P. G.; Allen, A. J.; Jemian, P. R. Glassy Carbon as an Absolute Intensity Calibration Standard for Small-Angle Scattering. *Metall. Mater. Trans. A* **2010**, *41*, 1151–1158.

(30) Lee, K. K.; Tsuruta, H.; Hendrix, R. W.; Duda, R. L.; Johnson, J. E. Cooperative Reorganization of a 420 Subunit Virus Capsid. *J. Mol. Biol.* **2005**, *352*, 723–735.

(31) Panda, D.; Datta, A. Implications of Area Normalization of Multi-Component Spectra. *Appl. Spectrosc.* **2008**, *62*, 341–344.

(32) Ferretti, R.; Zhang, J. W.; Buffle, J. Flocculation of Hematite with Polyacrylic Acid: Fractal Structures in the Reaction- and Diffusion-Limited Aggregation Regimes. *J. Colloid Interface Sci.* **1998**, *208*, 509–517.

(33) Teixeira, J. Small-Angle Scattering by Fractal Systems. *J. Appl. Crystallogr.* **1988**, *21*, 781–785.

(34) Lin, M. Y.; Lindsay, H. M.; Weitz, D. A.; Ball, R. C.; Klein, R.; Meakin, P. Universality in Colloid Aggregation. *Nature* **1989**, *339*, 360–362.
